# Effect of evidence-based therapy for secondary prevention of cardiovascular disease: Systematic review and meta-analysis

**DOI:** 10.1371/journal.pone.0210988

**Published:** 2019-01-18

**Authors:** Tian-Tian Ma, Ian C. K. Wong, Kenneth K. C. Man, Yang Chen, Thomas Crake, Muhiddin A. Ozkor, Ling-Qing Ding, Zi-Xuan Wang, Lin Zhang, Li Wei

**Affiliations:** 1 Research Department of Practice and Policy, UCL School of Pharmacy, London, United Kingdom; 2 Centre for Safe Medication Practice and Research, Department of Pharmacology and Pharmacy, Li Ka Shing Faculty of Medicine, The University of Hong Kong, Hong Kong, China; 3 UCL Institute of Cardiovascular Science, Univeristy College London, London, United Kingdom; 4 Barts Heart Centre, St. Bartholomew’s Hospital, West Smithfield, London, United Kingdom; 5 Department of Pharmacy, The Affiliated Cardiovascular Hospital of Xiamen University, Xiamen, China; 6 Intensive Care Unit, The First Affiliated Hospital of University of Science and Technology of China, Hefei, China; University of Tampere, FINLAND

## Abstract

**Background:**

The combination pharmacotherapy of antiplatelet agents, lipid-modifiers, ACE inhibitors/ARBs and beta-blockers are recommended by international guidelines. However, data on effectiveness of the evidence-based combination pharmacotherapy (EBCP) is limited.

**Objectives:**

To determine the effect of EBCP on mortality and Cardiovascular events in patients with Coronary Heart Disease (CHD) or cerebrovascular disease.

**Methods:**

Publications in EMBASE and Medline up to October 2018 were searched for cohort and case-control studies on EBCP for the secondary prevention of cardiovascular disease. The main outcomes were all-cause mortality and major cardiovascular events. Meta-analyses were performed based on random effects models.

**Results:**

21 studies were included. Comparing EBCP to either monotherapy or no therapy, the pooled risk ratios were 0.60 (95% confidence interval 0.55 to 0.66) for all-cause mortality, 0.70 (0.62 to 0.79) for vascular mortality, 0.73 (0.64 to 0.83) for myocardial infarction and 0.79 (0.68 to 0.91) for cerebrovascular events. Optimal EBCP (all 4 classes of drug prescribed) had a risk ratio for all-cause mortality of 0.50 (0.40 to 0.64). This benefit became more dilute as the number of different classes of drug comprising EBCP was decreased—for 3 classes of drug prescribed the risk ratio was 0.58 (0.49 to 0.69) and for 2 classes, the risk ratio was 0.67 (0.60 to 0.76).

**Conclusions:**

EBCP reduces the risk of all-cause mortality and cardiovascular events in patients with CHD or cerebrovascular disease. The different classes of drugs comprising EBCP work in an additive manner, with optimal EBCP conferring the greatest benefit.

## Introduction

Cardiovascular disease (CVD) is the leading cause of mortality and morbidity worldwide. Based on statistics from The World Health Organization (WHO), coronary heart disease (also known as ischaemic heart disease) and stroke are the top two causes of death globally [1]. Pharmacological therapy plays a key role in the secondary prevention of CVD. Large evidence supports drugs conferring mortality benefit from several different classes: antiplatelet agents, angiotensin-converting enzyme inhibitors (ACEIs)/angiotensin receptor blockers (ARBs), beta blockers and lipid-lowering drugs [2–4]. These are recommended by the WHO [5] and guideline bodies including the National Institute for Health and Care Excellence (NICE) [6,7], the European Society of Cariology (ECS) [8], the American College of Cardiology/American Heart Association (ACC/AHA) [9] and American Heart Association/American Stroke Association (AHA/ASA) [10].

In 2001, a fix-dose combination pill was proposed by the WHO[5] and was specified as a combination of aspirin, beta-blocker, ACEI and statin. In 2003, Wald and Law proposed that a fixed-dose combination pill, called polypill, consisting of a statin, BP-lowering agents, aspirin and folic acid, could potentially reduce the risk of CVD by 80% in individuals from age 55[11]. Since the concept was presented, many research studies investigated the efficacy of different medication combinations. A recent systematic review and meta-analysis summarized 13 randomized controlled trials (RCTs) of different polypills with a total n = 9059, mainly conducted in individuals with pre-existing atherosclerotic cardiovascular disease. The relatively short duration of follow-up meant that there were no definitive conclusions possible supporting mortality benefit of polypill from the RCT level evidence. [12]. The current RCTs focused on comparison between polypill and usual care. There is still lack of RCT-level evidence on the effectiveness of individual drug combinations. The existing evidence on individual drug combinations is from some previous observational studies, which have examined the impact of the combination of antiplatelet agents, ACEIs/ARBs, beta-blockers and lipid-modifiers, called evidence-based combination pharmacotherapy (EBCP) [13–17], but there has been no systematic review to synthesize these together.

Uncertainties surrounding EBCP that have not yet been systematically assessed include: (i) whether there is conclusive statistical evidence suggesting multi-drug treatments do better than single-drug treatments for mortality benefit (ii) whether increasing the number of components will confer additional benefits; and (iii) the role of each component of combination therapy, and whether certain combinations have more potent mortality lowering effects. This systematic review was conducted with a meta-analysis of existing observational studies that investigated the impact of the EBCP on mortality and cardiovascular events in the secondary prevention of CVD.

## Methods

The Preferred Reporting Items for Systematic Reviews and Meta-Analysis (PRISMA) statement was used to guide the reporting of the methods and findings.[18,19]. A completed PRISMA checklist is provided as an additional file (S1 Appendix). The study protocol was registered in the International Prospective Register of Systematic Reviews database (PROSPERO: CRD42018078069).

### Systematic literature search

We performed the systematic literature search without limitations of language on EMBASE (1980 to October 2018) and Medline (1946 to October 2018). The search strategies were developed based on the PICO (population, intervention, comparator and outcome) principle[20], search terms (S2 Appendix) covering CVD (coronary heart disease (CHD) and stroke), cardiovascular drugs (lipid-modifiers, antiplatelet agents and first-line antihypertensive drugs) and terms for combination therapy. We also examined the bibliographies of some relevant reviews and articles to identify any additional studies.

### Study selection

Three investigators (TTM, ZXW and LZ) independently screened studies to be included in the review using predetermined inclusion criteria. Studies were included in the systematic review if they: (i) included participants aged ≥18 years old with a history of coronary heart disease (MI, stable or unstable angina pectoris), stroke or transient ischaemic attack (TIA); (ii) clearly defined exposure to a combination pharmacotherapy including at least one antiplatelet agent, one lipid-modifier and one drug of ACEI/ARB, beta-blockers or other commonly used cardiovascular drugs (diuretics, calcium channel blockers, α-adrenergic blockers, aldosterone antagonist, or renin inhibitor); (iii) clearly defined the outcome of all-cause mortality, major cardiovascular events (fatal or non-fatal MI, angina, stroke or TIA); (iv) reported relative risk/risk ratio (RR), hazard ratio (HR) or odds ratios (OR) or provided data for calculating the risk estimates.

There was no restriction on sample size or language. Conference proceedings and abstracts were excluded if there was insufficient data for determining the risk estimates and the 95% confidence intervals (CI); or if they were not cohort or case-control studies.

Antiplatelet agents included: acetylsalicylic acid, adenosine reuptake inhibitors, adenosine diphosphate receptor inhibitors, and P2Y12 antagonists. Lipid-modifiers consisted of all statins, bile acid sequestrants, ezetimibe, fibrates and nicotinic acid. Other commonly used cardiovascular drugs included thiazide-type diuretics, loop diuretics, aldosterone antagonists, calcium channel blockers (CCBs), α-adrenergic blockers and renin inhibitors.

### Appraisal of study quality

Two investigators (TTM and LQD) independently assessed the methodological quality of included observational studies reviewing the study design, implementation, loss to follow-up, exposure and outcome determination. We adapted the Newcastle-Ottawa Scale (NOS)[21] for assessing the quality of the included studies. Separate NOS criteria were used for case-control and cohort studies. Each version has eight items within three domains with a maximum of nine stars (*): selection (representativeness), comparability (due to design or analysis), and outcomes (assessment and follow-up). A study can receive one star for meeting each criterion, while a maximum of two stars can be given for comparability (design or analysis). Studies with one star for comparability only controlled for age and gender in the analysis whereas studies with two stars under comparability also controlled for other important variables such as body mass index, comorbidity, laboratory tests or use of other relevant drugs. A final score ≥ seven was considered as high quality[22].

### Data extraction and management

Authors TTM and LQD independently completed the data extraction form which was cross-matched to ensure consistency and accuracy. Details of the study duration and design, sample size and participant characteristics, study setting and data source, intervention(s) and outcome(s) definitions, covariates from each of the included studies were extracted. Risk estimates in the form of RR, OR or HR and their corresponding 95% CIs were used as a measure of the association between intervention and outcome. For each study, we extracted the risk estimates adjusted for the most number of confounding variables. For studies without an adjusted result, the crude results were used for analysis.

### Data analysis

The risk estimates of each observational study were pooled in the meta-analysis to obtain the pooled RR. When a single study presented several risk estimates (i.e., separate estimates for the combination of four and three drugs), we adjusted the pooled estimates for within-study correlation. The inverse variance method with random effects models was used to calculate the pooled RRs and 95% CIs[23].

Heterogeneity was assessed using the Cochran Q test and Higgins’ I^2^ statistic[19]. Galbraith plot and subgroup analyses were carried out to investigate potential sources of heterogeneity and conduct sensitivity analyses. Galbraith plot evaluates the weight of each study on the meta-analysis by estimating the average RR and its contribution to the Q test[24]. In sensitivity analyses, we excluded studies with high weight shown by Galbraith plot and repeated the random-effects meta-analysis. Subgroup analyses were conducted to identify study-level heterogeneous factors, which included design (prospective cohort study, retrospective cohort study and case-control study), diagnosis of CVD (CHD, acute coronary syndrome (ACS), MI and stroke), age (<65 years, 65–75 years and >75 years), length of follow-up (<1 year, 1 year and >1 year), study regions (Europe, Asia, North America, multi-regions) and different treatment groups. All statistical analyses were performed using STATA version 15.0 and Revman version 5.3.

## Results

### Results from systematic literature search

**Fig 1:**
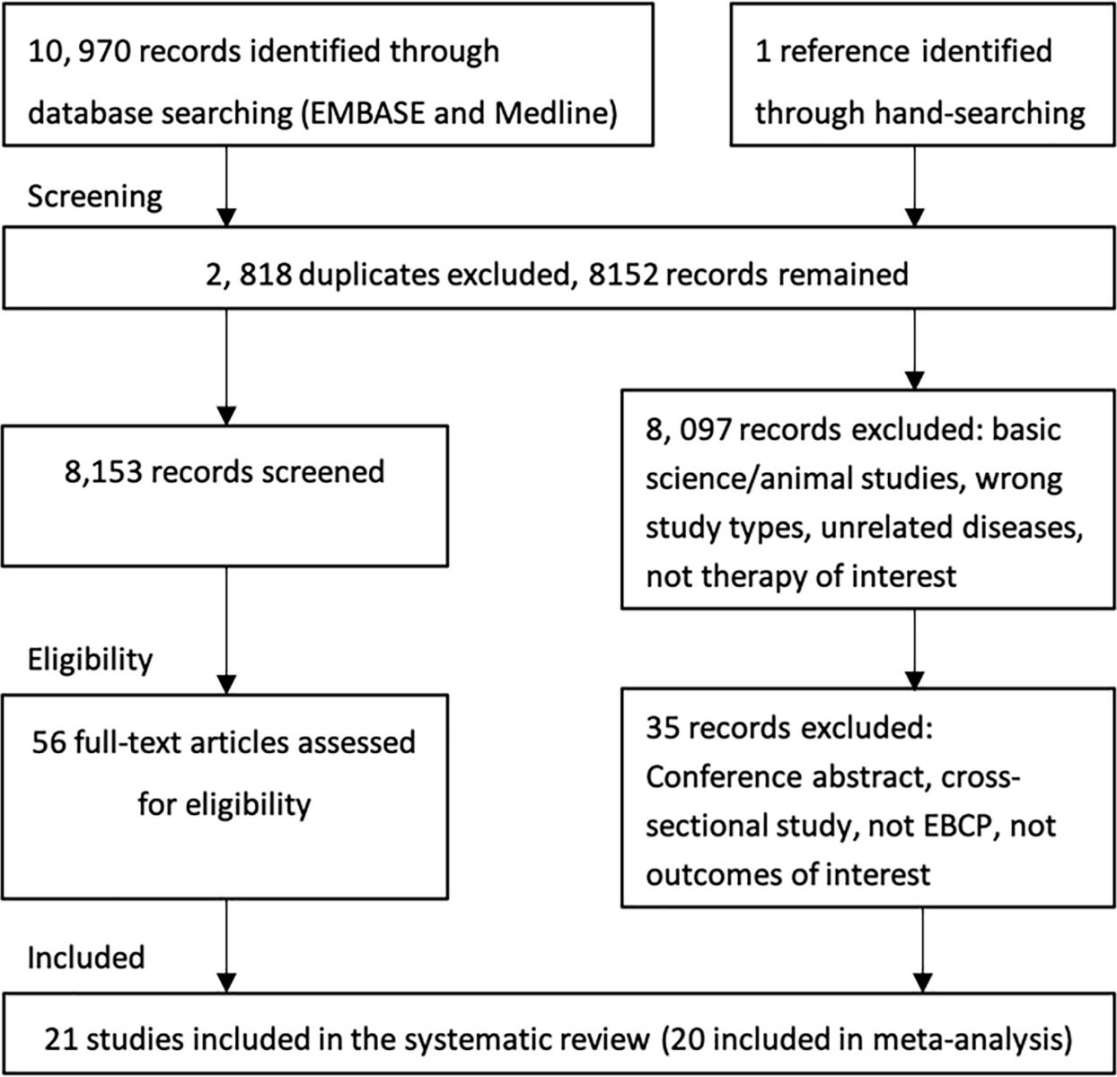
PRISMA flow chart summarising study identification and selection.

A total of 10,970 records were exported from the literature research. Titles and abstracts were screened and the full texts of 56 articles were further reviewed. 21 studies met the inclusion criteria for this systematic review, involving 117,881 participants with CVD. Fig 1 shows our search and selection process.

### Characteristics and quality of included studies

Table 1 summarize the characteristics of the included studies. All studies were published in English and from 2005 onwards: twelve were prospective cohort studies, six were retrospective cohort studies[13–17,25–37], and three were case-control studies[38–40]. Twenty observational studies included were considered as high quality according to their NOS score ≥ seven (S2 and S3 Tables). The study of Timoteo et al.[32] was excluded due to the low quality with a NOS score of five.

**Table 1:** Characteristics of included studies.

Author, year	Study design	Country	Inclusion Criteria	No. of Participants	Mean Age ± SD (Range)	Study duration	Medications	Exposure ascertainment	Outcome assessment
Al-Zakwani 2012	Prospective cohort study	6 Middle Eastern countries	Consecutive patients hospitalized with ACS	7567	56 ± 12	1 year	Combination of antiplatelet beta-blocker, ACEI/ARB and statin	Structured interview; discharge drugs	Telephone interviews
Amann 2014	Prospective cohort study	Germany	Consecutive patients hospitalized for an AMI	3844	62 (28–74)	6 years	Combination of antiplatelet beta-blocker, ACEI/ARB and statin	Structured interview; discharge drugs	German population-based AMI registry; structured interview
Bauer 2010	Prospective cohort study	Germany	Consecutive hospital survivors of AMI	11823	Group 1: 71.1 (61.8–79) Group 2: 65.0 (56.0–73.4)	1 year	ASA, clopidogrel, bata-blocker, ACEI/ARB and statin	Structured interview; discharge drugs	Structured interview following determined criteria
Bezin 2017	Retrospective cohort study	France	Patients hospitalised for an ACS; aged ≥20 years	2874	67 (56–77)	3.6 years (2.2–5.3)	Bata-blockers, antiplatelet agents, statins and ACEI/ARB	EGB database; ATC code; exposure defined according to drug dispensing in the 3-month period following initial ACS	EGB database; ICD-10 cades
Bramlage 2010	Prospective cohort study	Germany	Consecutive patients hospitalized for an AMI	5353	EBCP: 66.3 (56.9–75.1) Sub-EBCP: 70.5 (60.9–79.1)	1 year	Combination of ACEI/ARB, beta-blocker, statins, aspirin, clopidogrel unless contraindicated	Structured interview; secondary prevention at hospital discharge	SAMI registry; structured interview following determined criteria
Chen 2017	Retrospective cohort study	China	CAD patients	3176	EBCP: 64.4 Non-EBCP: 64.4	27.1 months	Combination of antiplatelet agents, statins, beta-blockers and ACEI/ARB	Medical records; discharge drugs	CAD database of West China hospital; identified with determined criteria; followed telephone or hospital-visits
Danchin 2005	Prospective cohort study	France	Consecutive patients with AMI	2119	Triple therapy: 71 (58–79) Non-triple therapy: 62 (51–72)	1 year	Combination of antiplatelet agents, beta-blocker and statins	Structured interview; discharge drugs	Structured interview
Gouya 2007	Retrospective cohort study	Austria	Patients with AMI	250	70 ± 14 (34–93)	552±200 days	ACEI/ARB, beta-blockers, antiplatelet agents and lipid-lowering agents	BGKK database; ATC codes; discharge drugs	BGKK database; ICD-9 codes
Gunnel 2013	Retrospective cohort study	Australia	Patients hospitalized for a first AMI	9580	Hierarchy	11 years	Bata-blockers (BB), statins (ST) and ACEI/ARB	PBS register; PBS item codes; drugs received during the 29-day exposure period post-discharge for the primary AMI	Hospital morbidity data collection; Mortality Register; ICD-9 codes
Kopel 2014	Prospective national cohort study	USA	Hospital survivors of ACS	9107	1 drug: 67 ± 14 2 drugs: 65 ± 14 3 drugs: 63 ± 13 4 drugs: 63 ± 12	1 year	Antiplatelet, beta-blockers, statins, ACEI/ARB	Structured interview; discharge drugs	ACS Israeli Survey; National Population Registry; computerized audit checks and queries
Lafeber 2013	Prospective cohort study	Netherlands	Patients with CAD	2706	60 ± 9	5.0 years (2.4–10.2)	Aspirin, statins, BP-lowering agents	Structured interview	Structured interview
Lee 2010	Prospective cohort study	Korea	Hospital survivors of AMI	9294	63.8 ± 12.5	180 ± 35 days	Combination of antiplatelet agents, statins, beta-blockers and ACEI/ARB	Structured interview; discharge drugs	KAMIR registry; medical records; telephone interview
Mukherjee 2004	Prospective cohort study	USA	Patients with ACS	1358	63.7 ± 13.3	6 months	Antiplatelet drugs, BB, ACEI and lipid-lowering agents	Structured interview; discharge drugs	Health system record review or phone call interview
Park 2015	Retrospective cohort study	USA, Canada and Scotland	Non-cardioembolic stroke patients aged ≥ 35 years old	3680	Level 0: 63.3 ± 11.5 Level 1: 65.6 ± 12.5 Level 2: 67.2 ± 11.1 Level 3: 65.7 ± 10.2	2 years	Antihypertensive agents, lipid modifiers and antithrombotic agents. Composite appropriateness level: level 0, none of the indicated medications prescribed; level 1, 1 medication prescribed even though 3 medications indicated; level 2, 2 medications prescribed even though 2 medications indicated; and level 3, all indicated medications were prescribed.	Data from VISP trial; structured interview	Data from VISP trial; structured interview
Tay 2005	Prospective cohort study	Singapore	Consecutive patients with confirmed MI	5529	Young: 57 ± 10.7 Elderly: 81.42 ± 5.3	1 year	Antiplatelet agents, beta-blockers, ACEI/ARB, lipid-lowering agents	Structured interview; discharge drugs	Structured interview
Timoteo 2006	Retrospective cohort study	Portugal	Consecutive patients hospitalized for ACS	368	65 ± 13	30 days	Antiplatelet agents, beta-blockers, ACEI, statins	Hospital clinical data; drugs at discharge or of and event, whichever occurred first	Hospital clinical data or telephone contact
Yan 2007	Prospective cohort study	Canada	Patients with ACS	5833	65 (55, 74)	1 year	Combination of antiplatelet/anticoagulant, beta-blocker, ACEI and lipid-modifying therapies	Structured interview; discharge drugs	Canadian ACS Registry; structured interview; telephone interview
Zeymer 2011	Prospective cohort study	Germany	Patients with AMI and treated with a beta-blocker at discharge	9998	0–1 drug: 70.1 (60.3, 78.0) 2 drugs: 67.6 (58.2, 76.3) 3 drugs: 64.7(55.5, 73.0)	396 days	Aspirin, ACEI and statins	Structured interview; discharge drugs	ACOS registry; structured interview
Hippisley 2005	Nested case-control study	UK	Patients with a fist diagnosis of ischaemic heart disease	13029	Cases: 80 (73, 86) Controls: 80 (73, 85)	Cases: 20.3 months; controls: 21.0 months	Different combinations of statins, aspirin, beta-blocker and ACEI	Medical records	QRESEARCH database
Kirchmayer 2013	Nested case-control study	Italy	Patients with a diagnosis of AMI; aged 35–100 years	6880	Women: 72.5 Men: 63.7	994.5 days	Combination of antiplatelet agents, beta-blockers, statins and ACEI/ARB	Regional registry; ACT classification system	Data from the HIS; regional MIS database; ICD-9-CM codes
Van 2007	Nested case-control study	Netherlands	Patients with a history of MI	3513	Cases: 66.8 Controls: 66.0	Cases: 32.6 months; controls: 30.7 months	Different combinations of statins, antiplatelet agents, beta-blocker and ACEI	Medical records	PHARMO record linkage system; ICD-9-CM codes

Abbreviations: ACEI = angiotensin-converting enzyme inhibitor; ACOS = Acute Coronary Syndromes; ACS = acute coronary syndromes; AMI = acute myocardial infarction; ARB = angiotensin receptor blocker; ASA = acetyl salicylic acid; ATC = the Anatomical Therapeutic Chemical; BGKK = Burgenländische Gebietskrankenkasse; CAD = coronary artery disease; EBC = evidence-based component EGB = Echantillon Généraliste de Bénéficiaires; ICD = International Classification of Disease; KAMIR = the Korea Acute Myocardial Infarction Registry; MI = myocardial infarction; No. = number; OMT = optimal medical therapy; PBS = Pharmaveutical Benefits Scheme; SAMI = secondary prevention after acute myocardial infarction; SD = standard deviation; USA = the United States of America; VISP = Vitamin Intervention for Stroke Prevention

### Mortality

**Fig 2:**
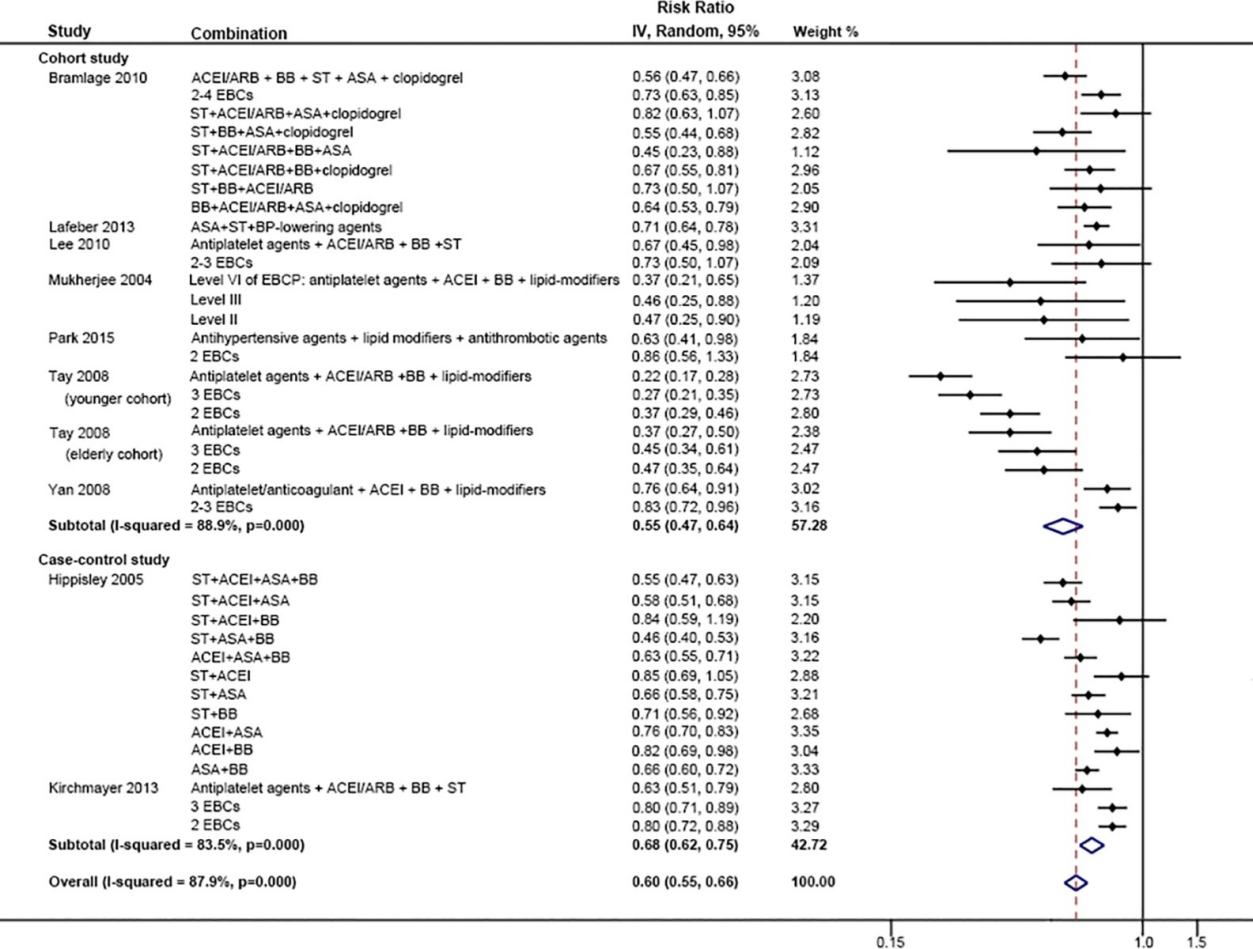
Comparison: EBCP versus 0–1 EB component, Outcome: All-cause mortality.

We included seven cohort and two case-control studies that provided results from combinations of EBCP and compared the risk of all-cause mortality with none or one component of EBCP in the primary meta-analysis (Fig 2). All the included studies presented a potential benefit of combination therapy with a lower risk of all-cause mortality. The pooled RRs of cohort and case-control studies were 0.55 (95% CI 0.47–0.64) and 0.68 (95% CI 0.62–0.75) respectively. Overall, the use of combination therapy reduced the risk of all-cause mortality by 40% (95% CI 34%-45%). In the study of Tay *et al*. [28], the outcomes were examined between younger patients (age < 75 years) and elderly patients separately. Younger patients benefited more from combination therapy than elderly individuals.

**Fig 3:**
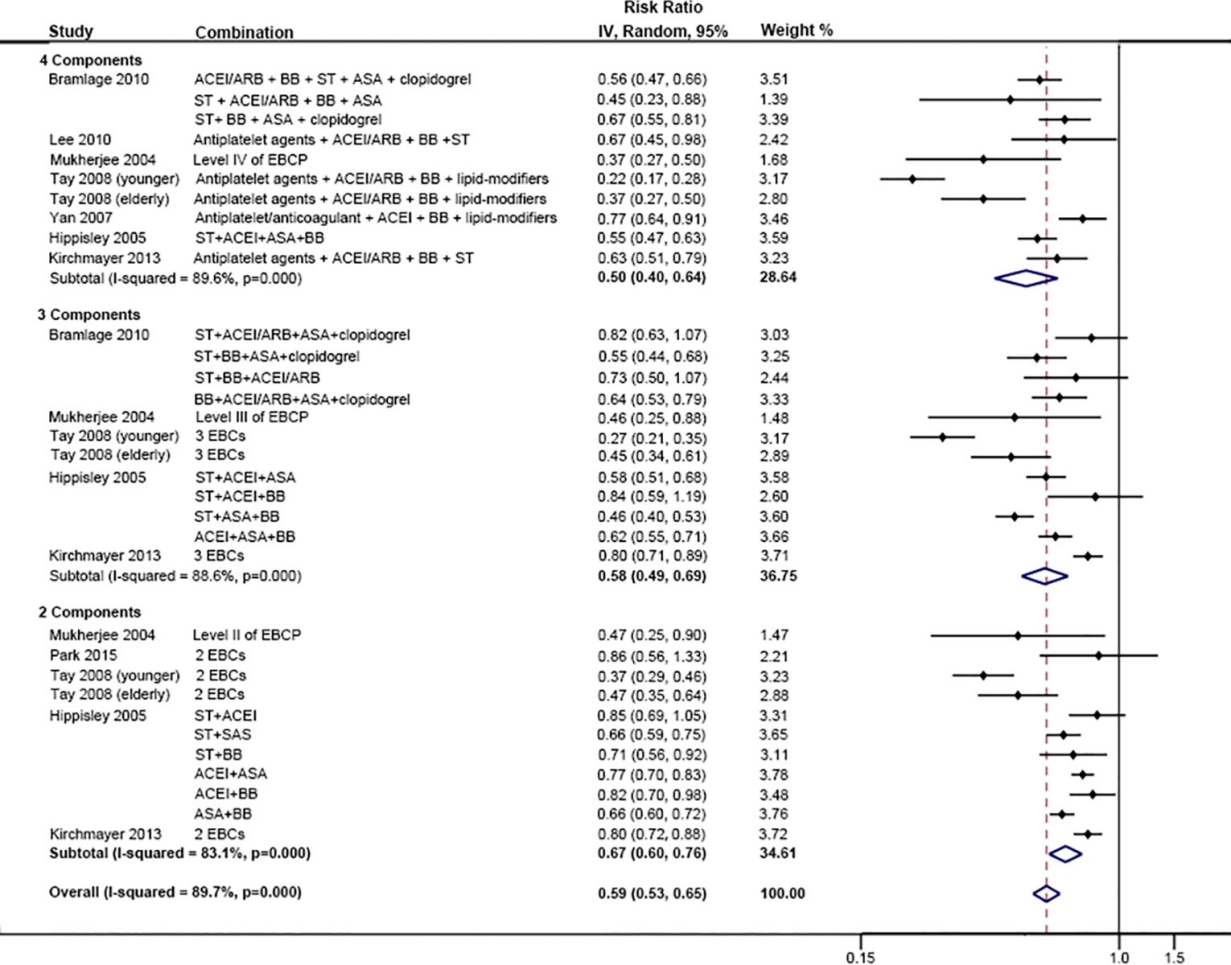
Comparison: Combination therapy of different numbers of components versus 0–1 component, Outcome: All-cause mortality.

**Fig 4:**
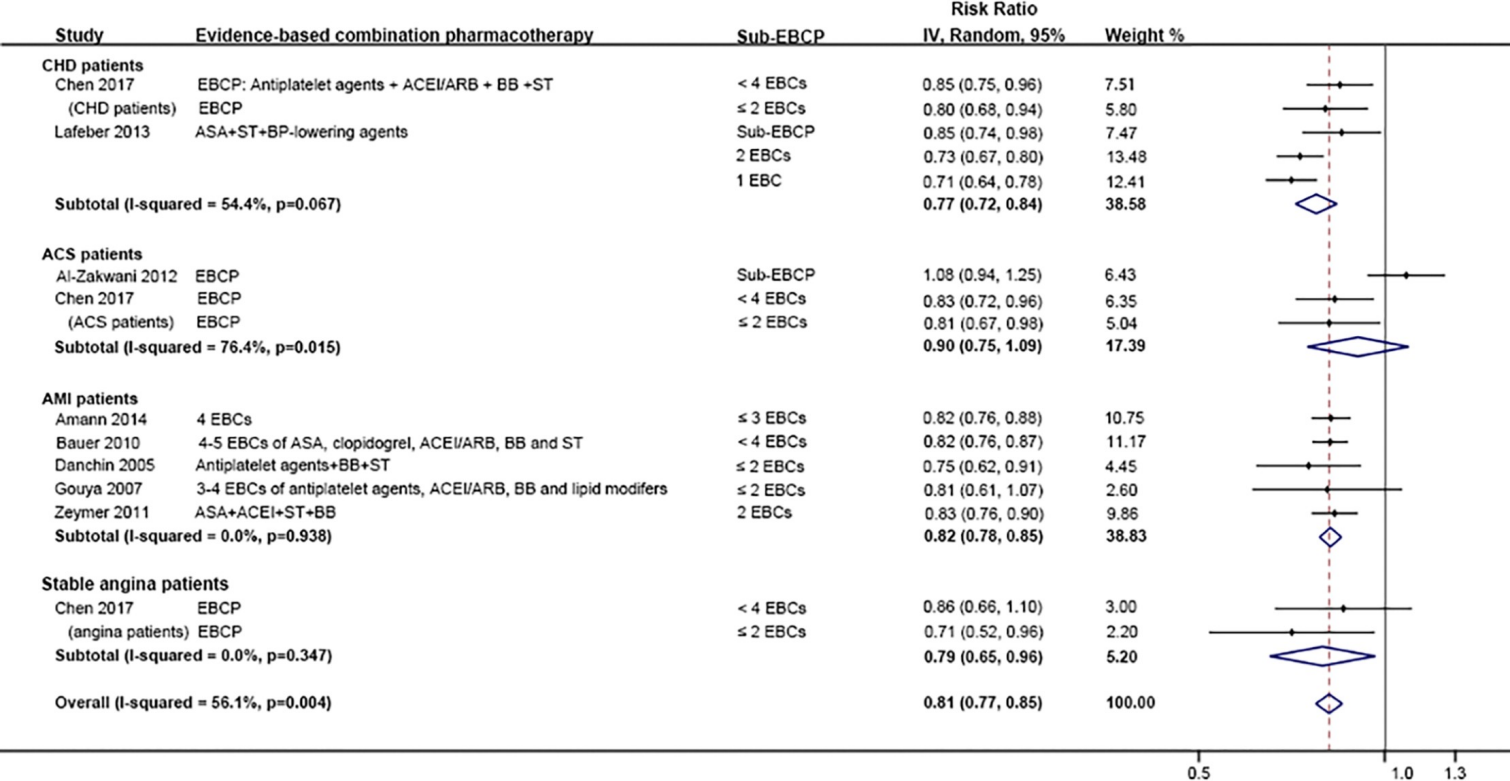
Comparison: EBCP versus sub-EBCP (< 4 components), Outcome: All-cause mortality.

Although we could not identify a statistically significant difference, the RRs of all-cause mortality improved with each additional component of EBCP added: 0.67 (95% CI 0.60–0.76), 0.58 (95% CI 0.49–0.69) and 0.50 (95% CI 0.40–0.64) in patients with two, three and four components respectively (Fig 3). Compared with suboptimal EBCP (less than 4 components), optimal EBCP was associated with a lower risk of all-cause mortality by 19% (95% CI 15%-23%) (Fig 4). The effects were similar in all patients with CHD (RR: 0.77, 95% CI 0.72–0.84), and subgroups of: angina (RR: 0.79, 95% 0.65–0.96), MI (RR: 0.82, 95% CI 0.76–0.88), and acute coronary syndromes 0.90 (95% CI 0.75–1.09) (Fig 4).

**Fig 5:**
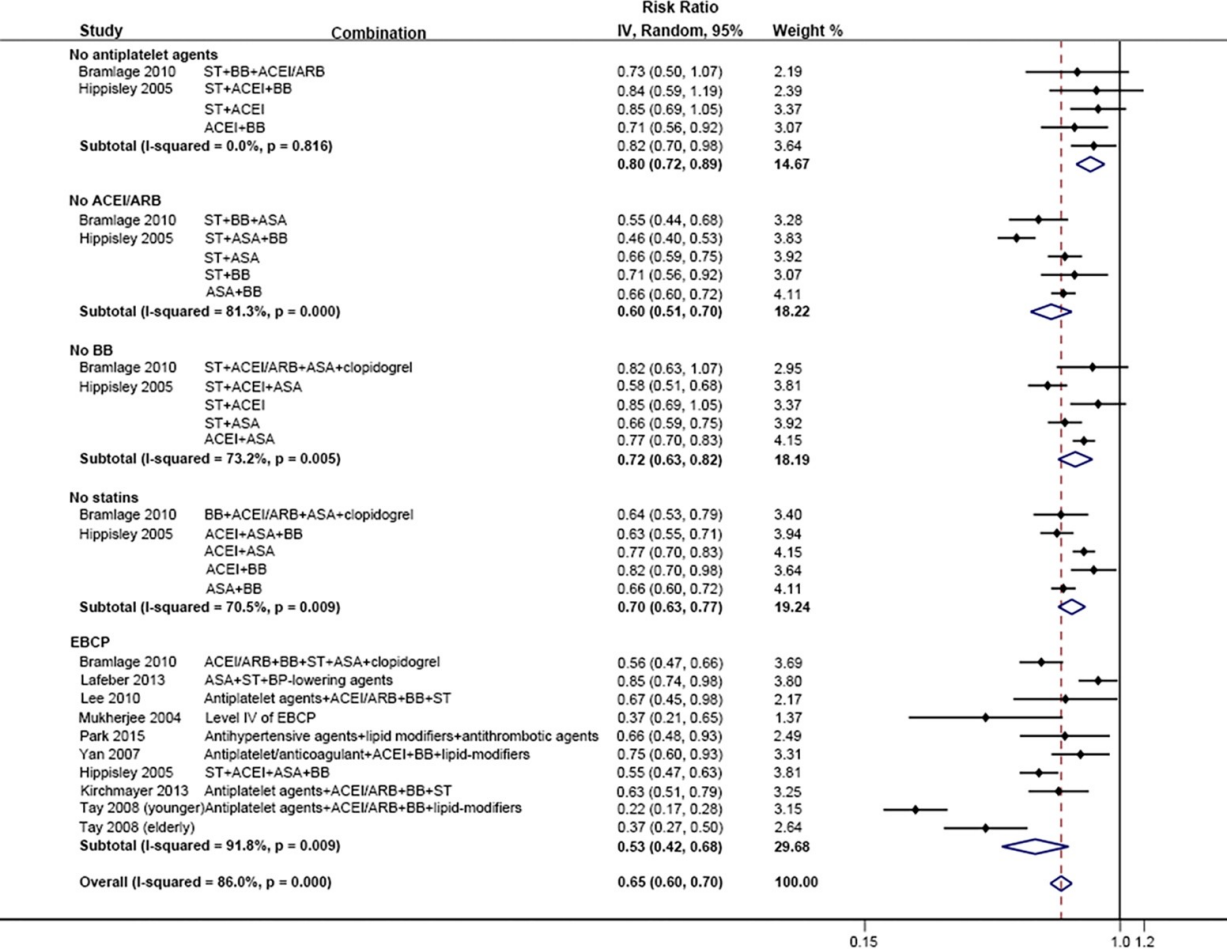
Comparison: Combination excluding one component versus 0–1 EB component, Outcome: All-cause mortality.

To assess the weight of each component of EBCP on outcomes, we evaluated pooled estimate effects of combination therapy excluding any one component (Fig 5). The results show that omitting any one component would reduce the potential beneficial effects of optimal EBCP (RR: 0.53, 95% CI 0.42, 0.68). The changes were greatest when excluding antiplatelet agents (RR: 0.80, 95% CI 0.72, 0.89). The difference was modest when omitting beta-blocker (RR: 0.72, 95% CI 0.63, 0.82) and statins (RR: 0.70, 95% CI 0.63, 0.77). The change of pooled estimate of omitting ACEI/ARB is shown to be inconspicuous (RR 0.60, 95% CI 0.51, 0.70).

### Major cardiovascular events

**Fig 6:**
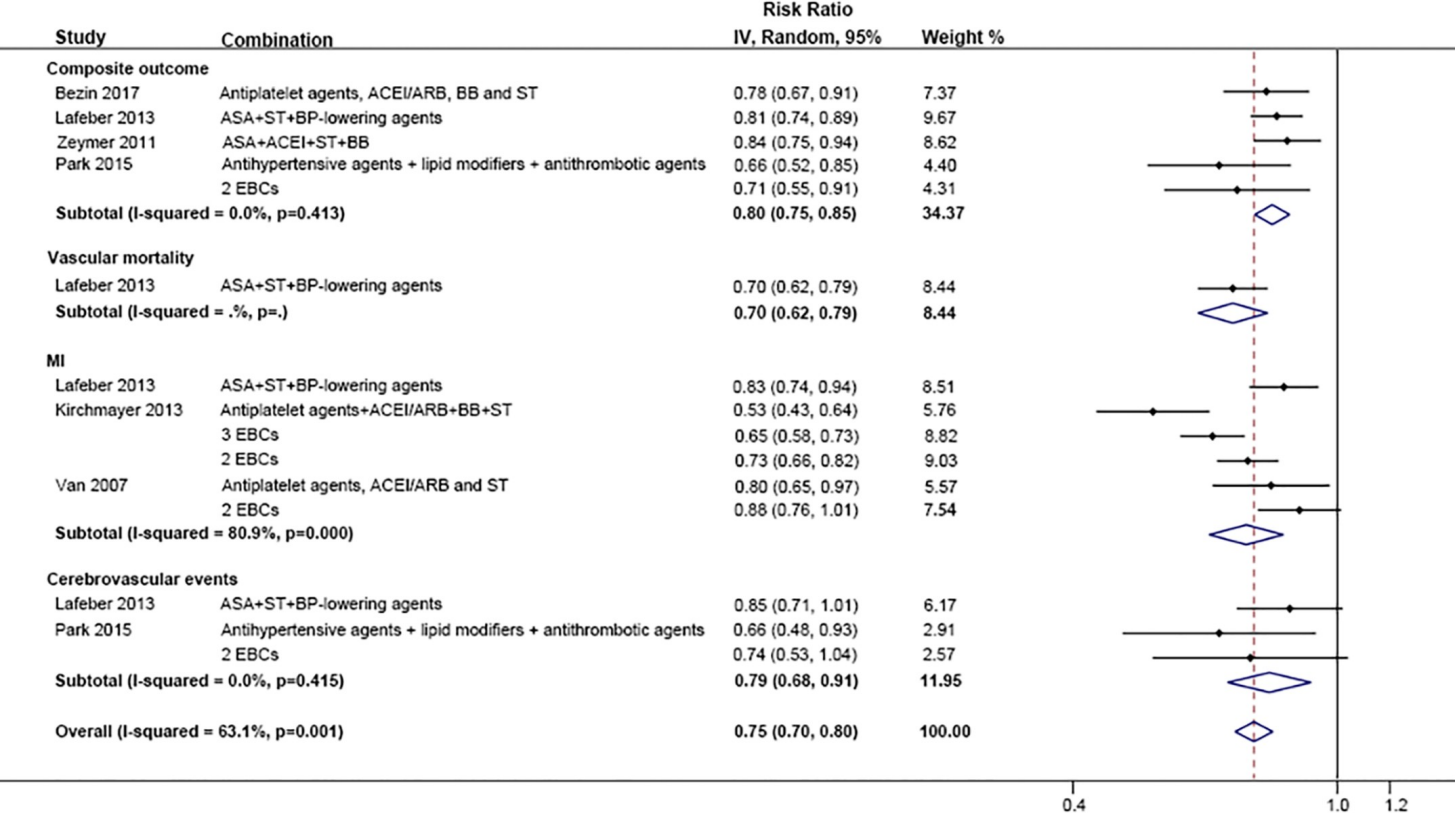
Comparison: EBCP versus 0–1 EB component, Outcome: Major CV events.

**Fig 7:**
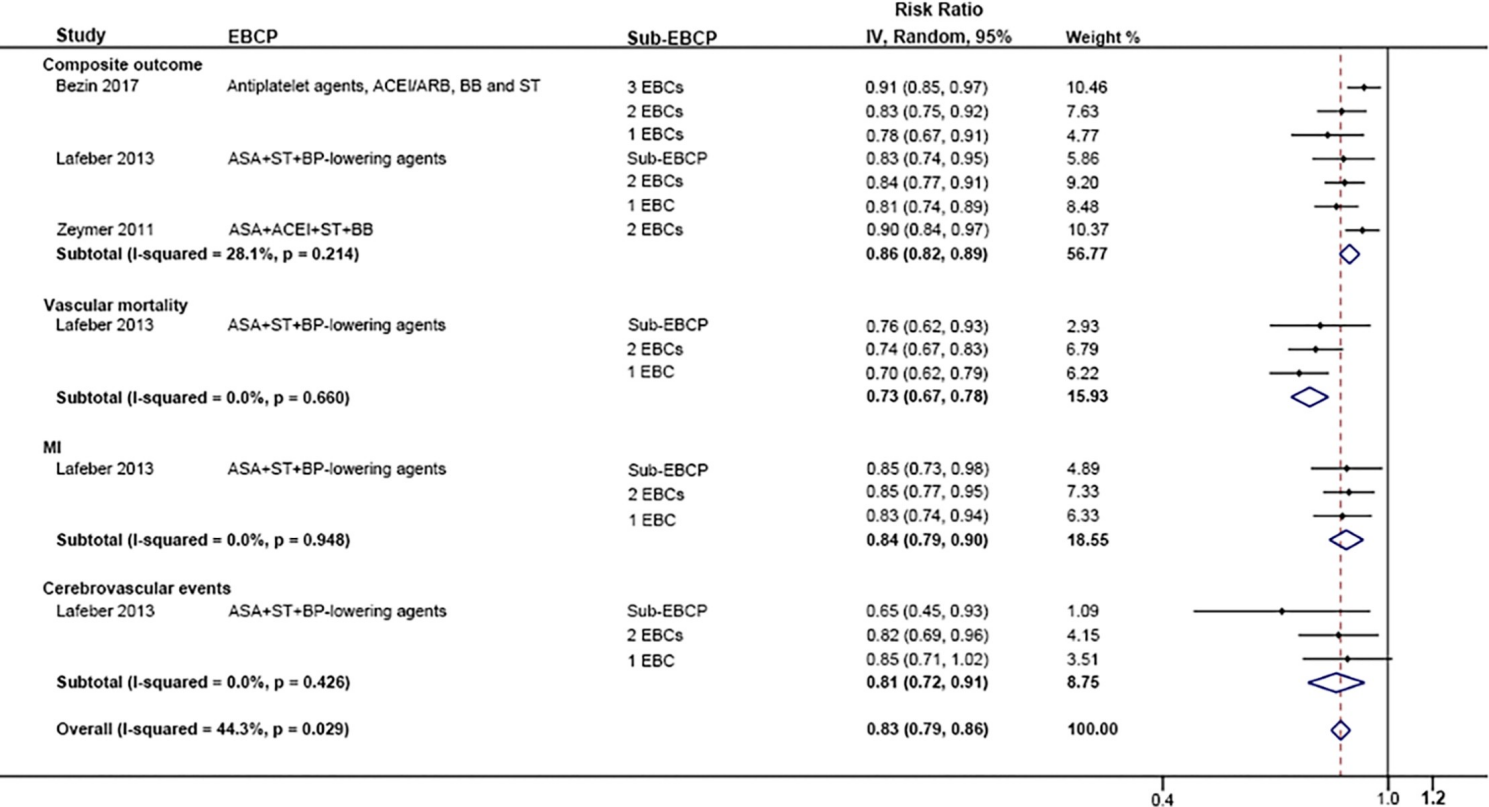
Comparison: EBCP versus sub-EBCP (< 4 components), Outcome: Subgroups of major CV events.

Three studies reported a composite outcome of mortality and major non-fatal cardiovascular events [16,25,27]. Compared with none or one component treatment, EBCP (>one drug) was associated with a lower risk of the composite outcome (RR: 0.80, 95% CI 0.75–0.85). Only Lafeber *et al*. reported the effect of combination therapy on the rate of vascular mortality, with an RR of 0.70 (95% CI 0.62, 0.79) [25]. The pooled result of Lafeber *et al*. [25], Kirchmayer *et al*. [39] and Van *et al*. [40] showed that combination treatment decreased the risk of MI by 28% (95% CI 17%-38%). Regarding cerebrovascular events, combination drug use also yielded a beneficial effect (RR: 0.79, 95% CI 0.68–0.91). In summary, compared with none or one EBCP component, the use of combination therapy reduced the relative risk of major cardiovascular events by 25% (95% CI 20%-30%) (Fig 6). Compared with suboptimal EBCP (less than 4 components), optimal EBCP was associated with a lower risk of cardiovascular events by 27% (95% CI 14%-21%) (Fig 7). The results present that optimal EBCP reduced the risk of composite outcome by 14% (95% CI 11%-18%), vascular mortality by 27% (95% CI 22%-33%), MI by 16% (95% CI 10%-21%) and cerebrovascular events by 19% (95% CI 9%-28%).

### Sensitivity analysis

The heterogeneity of the primary meta-analysis was high, with I^2^ = 87.9% (p < 0.001) (Fig 2). In the Galbraith plot (S3 Fig), the study of Tay et al. [28] induced the highest heterogeneity, followed by Hippisley *et al*. [38], Kirchmay *et al*. [39] and Yan *et al*. [30] We repeated the primary meta-analysis with the random-effects model after excluding each of the four studies (S4 Table). Tay *et al*. [28] was shown to be the largest contributor to heterogeneity. When omitting the study, I^2^ decreased to 72.1% though the pooled RR did not change remarkably (0.68, 95% CI 0.67, 0.72).

We undertook subgroup analyses to examine the potential sources of heterogeneity related to age, study regions, different diagnoses, length of follow-up and study designs on the EBCP’s effect on all-cause mortality (S5 Table). The results show significant differences between subgroups in age (P < 0.05), region (P < 0.01), follow-up duration (P < 0.05) and study type (P < 0.05), indicating the four covariates were likely to be associated with heterogeneity. Conversely, diagnosis of CVD, did not affect heterogeneity of the primary meta-analysis (P = 0.16). The results of subgroups by age show that younger patients may benefit more from reductions in all-cause mortality from EBCP than elderly individuals, with RRs of 0.44 (95% CI 0.30, 0.63) in patients aged <65, and 0.71 (95% CI 0.65, 0.77) and 0.62 (95% CI 0.56, 0.69) for 65–75 and >75 years old respectively. In terms of the subgroup analyses between different regions, the relative risk of mortality was lower in Asian patients on EBCP (RR: 0.40, 96% CI 0.31, 0.53) than patients in Europe (RR 0.67, 95% CI 0.63, 0.72), Canada/USA (RR 0.65, 95% CI 0.51, 0.83) or multi-region of USA, Canada and Scotland (RR 0.74, 95% CI 0.54, 1.01). Besides, the differences between follow-up duration (RR: < 1 year: 0.57, 1 year: 0.52 and >1 year: 0.69) and study types (RR: retrospective cohort study: 0.74, prospective cohort study: 0.54 and case-control study: 0.68) were also presented to be related to the heterogeneity. In addition, we performed another sensitivity analysis within studies which had the reference group of 0 EBCP drug (S4 Fig). The results showed no significant different from the primary meta-analysis (Fig 2).

## Discussion

Our meta-analysis of observational studies assessed the effects of EBCP with antiplatelet drugs, ACEIs/ARBs, beta-blockers, and lipid-modifiers on mortality and major cardiovascular events in CVD patients. The results show a benefit for EBCP, suggesting an overall decrease in the risk of all-cause mortality (approximately by 40%) and cardiovascular events (25%-30%) compared to either monotherapy or no therapy.

In this systematic review, we examined the effects of increasing the number of components of EBCP. The results show that each additional component of EBCP could confer a potential 8–9% survival benefit of patients with CVD with a median follow-up of one year. When weighting the impact of each component, we found that antiplatelet agents made the greatest contribution to the beneficial effects of combination therapy on survival in patients with CHD. Excluding antiplatelet drugs from optimal EBCP decreased the beneficial effects by 27%. Our results are supported by a meta-analysis of 193 RCTs. Based on 9,605 deaths, the study reported that antiplatelet therapy produced a significant 15% reduction in vascular deaths (P<0.0001) and about one-sixth of all-cause mortality (P<0.0001). The study also provided strong evidence of benefit from antiplatelet therapy to major cardiovascular events (non-fatal MI, non-fatal stroke or vascular death) [41].

Therefore, unless there are contraindications, antiplatelet agents should be considered as the first component of EBCP in the secondary prevention of CHD.

The evidence available from the literature for beta blockers and statin therapy is equally as strong. A meta-analysis of 147 RCTs suggested that beta-blockers could reduce CHD events by 29% (RR 0.71, 95% CI 0.66, 0.78). Additional RCT studies have also shown that beta-blockers play an important role in reducing mortality and morbidity for up to a year after an MI [42]. A meta-analysis of 14 RCTs of statins also demonstrated that statins could reduce the risk of all-cause mortality by 12% and major vascular events by 21% [43]. Thus, beta-blockers and statins count as valuable components of the optimal EBCP for CVD.

In our systematic review, we found a more modest effect for ACEI/ARB as part of EBCP. In particular two studies have also shown that the inclusion of ACEI/ARB in combination with statins, antiplatelet agents and beta-blockers was associated with a lower risk of mortality [17,38].

In this systematic review, we found some research gaps in terms of EBCP in secondary prevention of CVD.

Firstly, most studies included in the systematic review are based on CHD patients. Only the study of Park *et al*. [27] was conducted in stroke patients. There is a paucity of evidence for the benefit of EBCP in reducing the mortality risk in stroke patients, even though stroke represents a significant proportion of all cardiovascular disease. Whilst co-morbidities and risk factors cluster together, there is still a lack of data regarding any potential mortality benefit of ACEI and beta-blocker in post-stroke patients who otherwise do not have an indication for their prescription. This should be a priority area for further research.

Secondly, even though we did not limit any other conditions co-existing with CVD in the study population, we could not find any studies specifically evaluating the effects of EBCP for secondary prevention of CVD in patients with comorbidities. Most of the studies included in our review adjusted the risk estimates with comorbidities. Thus we were unable to identify if the results are applicable equally in the presence of other conditions. Comorbidities are highly prevalent in patients with CVD. A Dutch nationwide study found the percentage of patients with comorbidity were 40% and 32% in coronary heart disease and cerebrovascular disease, respectively [44]. In the context of clinical and functional heterogeneity, CVD patients with different co-conditions may have different responses to pharmacotherapy. In addition, interactions between cardiovascular drugs and treatment for comorbidities also need attention. For example, some nonsteroidal anti-inflammatory drugs like ibuprofen and naproxen are known to interfere with the antiplatelet effects of aspirin [45,46] as well as affect renal function and hence handling of all components of EBCP, in particular ACEIs and ARBs.

Thirdly, most studies included in this systematic review only focused on the combination of aspirin, clopidogrel, beta-blockers, ACEIs/ARBs and statins, observational evidence for the combination of some other commonly used drugs is lacking. This may in part be due to a lack of mortality benefit for many of these drugs tested in randomized trials (e.g. diuretics, CCBs [47], and fibrates [48]), a lack of conclusive evidence of benefits for some drugs on the secondary prevention of CVD (e.g. spironolactone and eplerenone [49]), but may also be due to a lack of follow-up time for newer medications that have come to market e.g. sacubitril/ valsartan combination.

Finally, the length of follow-up in most of the included studies was less than one year, and only effects of drugs in discharge were examined without considering other important long-term effects. These include the possibility of sequential drug exchange or poor drug adherence. Only the study of Bezin *et al*. [16] reported the cumulated use of cardiovascular drugs, showing a persistent benefit of combination therapy and additionally reductive effects on the occurrence of major adverse cardiac events or mortality when increasing the number of components.

### Strengths and limitations

In the absence of RCTs, we did the systematic review of observational studies. Our study has several strengths. Firstly, we undertook extensive analysis in exploring potential variables that could affect the effects of secondary prevention for CVD, hence providing clinicians with an evidence base for their decision-making. Secondly, our results are robust and consistent, as shown by our extensive analyses by using influence analysis, subgroup analysis and sensitivity analysis.

There are some limitations in the current study. Firstly, the results of some subgroup analyses were not credible enough because only one study was included. Secondly, differences in study designs, exclusion criteria, control groups selection, duration of follow-up, exposure and outcome definitions, including covariates and analyses models can affect the accuracy of pooled estimates for both crude and adjusted RRs. Thirdly, several studies reported the estimated effect sizes with HRs and ORs instead of RRs, and the exact statistical method was not clearly described. We were not able to exclude the influence on results by combining these three types of estimates in the meta-analysis. The variability between studies was unavoidable, and the study conclusions should be evaluated alongside the reported heterogeneity. Nevertheless, we conducted sensitivity analyses to examine the impact of heterogeneity between studies and assessed of the potential causes of heterogeneity. In addition, as studies included in each meta-analysis were less than ten, we did not examine the publication bias [50,51]. Considering all included studies reported a positive effect of combination therapy only with a difference in the extent, therefore we think that important publication bias due to a preferential publication of large studies with positive findings has not occurred.

## Conclusions

In conclusion, our systematic review and meta-analysis suggest that in patients with CVD, EBCP can reduce the risk of all-cause mortality by approximately 40% and major cardiovascular events by 25%-30%. Antiplatelet agents, beta-blockers and statins could be considered as stable components of combination therapy in secondary prevention of CHD.

## Supporting information

S1 Appendix PRISMA 2009 checklist. (DOCX)

S2 Appendix Search strategy. (DOCX)

S1 Table Summary of results of included studies. (DOCX)

S2 Table Evidence quality assessment, cohort studies. (DOCX)

S3 Table Evidence quality assessment, case-control studies. (DOCX)

S4 Table Relative risk and heterogeneity after excluding studies. (DOCX)

S5 Table Subgroup analysis of demographics and study methodology for all-cause mortality (EBCP versus 0–1 component). (DOCX)

S1 Fig Comparison: EBCP versus 0 EB component, Outcome: All-cause mortality. (DOCX)

S2 Fig Comparison: EBCP versus sub-EBCP (< 4 components), Outcome: All-cause mortality. (DOCX)

S3 Fig Comparison: EBCP versus 0–1 EB component, Outcome: Major CV events. (DOCX)

S4 Fig Galbraith plot for heterogeneity in the primary meta-analysis. (DOCX)
