# Supplementary material for: Effect of evidence-based therapy for secondary prevention of cardiovascular disease: Systematic review and meta-analysis
Source: PLoS One. 2019 Jan 18;14(1):e0210988. doi: 10.1371/journal.pone.0210988 (PMC6338367; doi:10.1371/journal.pone.0210988)
Supplement: S2 Appendix — (DOCX) [file pone.0210988.s002.docx]

| **Database** |  |  | **Search terms** |
| --- | --- | --- | --- |
| **MEDLINE** |  |  | **Cardiovascular diseases** |
|  |  | 1 | exp cardiovascular disease/ |
|  |  | 2 | exp Coronary Disease/ |
|  |  | 3 | exp Myocardial Ischemia/ |
|  |  | 4 | exp heart disease/ |
|  |  | 5 | exp acute coronary syndrome/ |
|  |  | 6 | exp angina pectoris/ |
|  |  | 7 | exp myocardial infarction/ |
|  |  | 8 | (isch?emi* adj3 heart).tw. |
|  |  | 9 | (myocard* adj3 (infarct* or re?vascular* or ischemi* or ischaem*)).tw. |
|  |  | 10 | (coronary adj3 disease*).tw. |
|  |  | 11 | ((coronary or cardiovascular or ischemic) adj event*).tw. |
|  |  | 12 | (heart adj (disease* or attack* or infarct*)).tw. |
|  |  | 13 | (cardiac adj3 disease).tw. |
|  |  | 14 | (morbid adj5 (heart* or cardiovascula* or coronary* or isch?em* or myocard*)).tw. |
|  |  | 15 | angina or MI.tw. |
|  |  | 16 | chd or cad.tw. |
|  |  | 17 | exp Stroke/ |
|  |  | 18 | (stroke or stokes or cerebrovasc* or cerebral vascular or apoplexy or (brain adj2 accident*)).tw. |
|  |  | 19 | (brain* or cerebral or lacunar) adj2 infarct*).tw. |
|  |  | 20 | or/1-19 |
|  |  |  | **Cardiovascular drugs** |
|  |  | 21 | exp Hydroxymethylglutaryl-CoA Reductase Inhibitors/ |
|  |  | 22 | hydroxymethylglutaryl-CoA reductase inhibitor*.tw. |
|  |  | 23 | HMG CoA reductase inhibitor*.tw. |
|  |  | 24 | HMG Co A reductase inhibitor*.tw. |
|  |  | 25 | statin*.tw. |
|  |  | 26 | exp colesevelam/ |
|  |  | 27 | colestyramine/ |
|  |  | 28 | colestipol/ |
|  |  | 29 | ezetimibe/ |
|  |  | 30 | fibric acid derivative/ |
|  |  | 31 | nicotinic acid/ |
|  |  | 32 | (atorvastatin or cerivastatin or dalvastatin or fluindostatin or fluvastatin or lovastatin or pitavastatin or pravastatin or rosuvastatin or simvastatin or meglutol or mevinolin* or monacolin* or pravachol or lipex or lipitor or zocor or mevacor or lescol or baycol or bezafibrate or bezalip or ciprofibrate or modalim or clofibrate or fenofibrate or lipantil or supralip or gemfibrozil or lopid or procetofen or tocofibrte or transferal or theofibrate or etofylline clofibrate or duolip or acipimox or olbetam or nicotinic acid or niaspan).tw. |
|  |  | 33 | or/21-32 **[Lipid modifiers]** |
|  |  | 34 | exp Platelet Aggregation Inhibitors/ |
|  |  | 35 | [Adenosine Diphosphate/ai [Antagonists & Inhibitors]] |
|  |  | 36 | (antiplatelet agents* or anti-platelet agent*).tw. |
|  |  | 37 | (antiplatelet therap* or anti-platelet therap*).tw. |
|  |  | 38 | thrombocyte aggregation inhibit*.tw. |
|  |  | 39 | platelet aggregation inhibit*.tw. |
|  |  | 40 | (antithrombocytic agent* or anti-thrombocytic agent*).tw. |
|  |  | 41 | (antithrombocytic therap* or anti-thrombocytic therap*).tw. |
|  |  | 42 | adenosine diphosphate receptor inhibit*.tw. |
|  |  | 43 | (adenosine reuptake inhibit* or adenosine re-uptake inhibit*).tw. |
|  |  | 44 | (aspirin or acetylsalicylic acid or dipyridamole or eptifibatide  or ticlopidine or  clopidogrel or cilostazol or (P2Y12 adj2 antagonis*) or prasugrel or cangrelor or ticagrelor or elinogrel tirofiban or picotamide or ticlid or beraprost or aggrenox or ditazole).tw. |
|  |  | 45 | or/34-44 **[Antiplatelet agents]** |
|  |  | 46 | exp thiazides/ |
|  |  | 47 | exp sodium chloride symporter inhibitors/ |
|  |  | 48 | exp sodium potassium chloride symporter inhibitors/ |
|  |  | 49 | exp Mineralocorticoid Receptor Antagonists/ |
|  |  | 50 | ((loop or ceiling) adj diuretic?).tw. |
|  |  | 51 | aldosterone antagonist* |
|  |  | 52 | (amiloride or benzothiadiazine or bendroflumethiazide or bumetanide or chlorothiazide or cyclopenthiazide or furosemide or hydrochlorothiazide or hydroflumethiazide or methyclothiazide or metolazone or polythiazide or trichlormethiazide or veratide or thiazide? or torasemide or torsemide or chlorthalidone or chlortalidone or phthalamudine or chlorphthalidolone or oxodoline or thalitone or hygroton or indapamide or metindamide or spironolactone or eplerenone).tw. |
|  |  | 53 | or/46-52 **[Diur]** |
|  |  | 54 | exp angiotensin-converting enzyme inhibitors/ |
|  |  | 55 | angiotensin converting enzyme inhibit*.tw. |
|  |  | 56 | (ace adj2 inhibit*).tw. |
|  |  | 57 | acei.tw. |
|  |  | 58 | (alacepril or altiopril or ancovenin or benazepril or captopril or ceranapril or ceronapril or cilazapril or deacetylalacepril or delapril or derapril or enalapril or epicaptopril or fasidotril or fosinopril or foroxymithine or gemopatrilat or idapril or imidapril or indolapril or libenzapril or lisinopril or moexipril or moveltipril or omapatrilat or pentopril* or perindopril* or pivopril or quinapril* or ramipril* or rentiapril or saralasin or snitrosocaptopril or spirapril* or temocapril* or teprotide or trandolapril* or utibapril* or zabicipril* or zofenopril* or Aceon or Accupril or Altace or Capoten or Lotensin or Mavik or Monopril or Prinivil or Univas or Vasotec or Zestril).tw. |
|  |  | 59 | or/54-58 **[ACEI]** |
|  |  | 60 | renin/ai |
|  |  | 61 | (aliskiren or ciprokiren or ditekiren or enalkiren or remikiren or rasilez or tekturna or terlakiren or zankiren).tw. |
|  |  | 62 | renin inhibit*.tw. |
|  |  | 63 | or/60-62 **[RI]** |
|  |  | 64 | exp angiotensin receptor antagonist/ |
|  |  | 65 | (angiotensin adj3 (receptor antagon* or receptor block*)).tw. |
|  |  | 66 | arb?.tw. |
|  |  | 67 | (abitesartan or azilsartan or candesartan or elisartan or embusartan or eprosartan or forasartan or irbesartan or losartan ormilfasartan or olmesartan or saprisartan or tasosartan or telmisartan or valsartan or zolasartan).tw. |
|  |  | 68 | or/64-67 **[ARB]** |
|  |  | 69 | calcium channel blocking agent/ |
|  |  | 70 | (amlodipine or aranidipine or barnidipine or bencyclane or benidipine or bepridil or cilnidipine or cinnarizine or clentiazem or darodipine or diltiazem or efonidipine or elgodipine or etafenone or fantofarone or felodipine or fendiline or flunarizine or gallopamil or isradipine or lacidipine or lercanidipine or lidoflazine or lomerizine or manidipine or mibefradil or nicardipine or nifedipine or niguldipine or nilvadipine or nimodipine or nisoldipine or nitrendipine or perhexiline or prenylamine or semotiadil or terodiline or tiapamil or verapamil or Cardizem CD or Dilacor XR or Tiazac or Cardizem Calan or Isoptin or Calan SR or Isoptin SR Coer or Covera HS or Verelan PM).tw. |
|  |  | 71 | (calcium adj2 (antagonist? or block* or inhibit*)).tw. |
|  |  | 72 | or/69-71 **[CCB]** |
|  |  | 73 | exp adrenergic beta-antagonists/ |
|  |  | 74 | (acebutolol or adimolol or afurolol or alprenolol or amosulalol or arotinolol or atenolol or befunolol or betaxolol or bevantolol or bisoprolol or bopindolol or bornaprolol or brefonalol or bucindolol or bucumolol or bufetolol or bufuralol or bunitrolol or bunolol or bupranolol or butofilolol or butoxamine or carazolol or carteolol or carvedilol or celiprolol or cetamolol or chlortalidone cloranolol or cyanoiodopindolol or cyanopindolol or deacetylmetipranolol or diacetolol or dihydroalprenolol or dilevalol or epanolol or esmolol or exaprolol or falintolol or flestolol or flusoxolol or hydroxybenzylpinodolol or hydroxycarteolol or hydroxymetoprolol or indenolol or iodocyanopindolol or iodopindolol or iprocrolol or isoxaprolol or labetalol or landiolol or levobunolol or levomoprolol or medroxalol ormepindolol ormethylthiopropranolol ormetipranolol ormetoprolol ormoprolol or nadolol or oxprenolol or penbutolol or pindolol or nadolol or nebivolol or nifenalol or nipradilol or oxprenolol or pafenolol or pamatolol or penbutolol or pindolol or practolol or primidolol or prizidilol or procinolol or pronetalol or propranolol or proxodolol or ridazolol or salcardolol or soquinolol or sotalol or spirendolol or talinolol or tertatolol or tienoxolol or tilisolol or timolol or tolamolol or toliprolol or tribendilol or xibenolol).tw. |
|  |  | 75 | (beta adj2 (adrenergic? or antagonist? or block* or receptor?)).tw. |
|  |  | 76 | or/73-75 **[BB]** |
|  |  | 77 | exp adrenergic alpha antagonists/ |
|  |  | 78 | (alfuzosin or bunazosin or doxazosin or metazosin or neldazosin or prazosin or silodosin or tamsulosin or terazosin or tiodazosin or trimazosin).tw. |
|  |  | 79 | (andrenergic adj2 (alpha or antagonist?)).tw. |
|  |  | 80 | ((andrenergic or alpha or receptor?) adj2 block*).tw. |
|  |  | 81 | or/77-80 **[AB]** |
|  |  | 82 | 53 or 59 or 63 or 68 or 72 or 76 or 81 |
|  |  | 83 | (33 and 45) or (33 and 82) or (45 and 82) |
|  |  | 84 | Evidence based.tw. |
|  |  | 85 | 83 or 84 |
|  |  |  | **Combination** |
|  |  | 86 | Drug Combinations/ |
|  |  | 87 | Drug treatment, combination/ |
|  |  | 88 | (polypill* or (drug* adj2 combin*) or ((multi* or several) adj2 (ingredient* or component* or therap* or treatment* or intervention*)) or policap or quintapill or (single adj2 pill* adj2 comb*) or single-pill or Red Heart pill*).tw. |
|  |  | 89 | (((mono* or single* or dual* or double* or triple*) adj3 (therap* or treatment* or intervention*)) or (intensive adj2 (lowing or reduction or management or therap* or treatment* or intervention*))).tw. |
|  |  | 90 | or/86-89 |
|  |  |  |  |
|  |  | 91 | 20 and 85 and 90 |
|  |  |  |  |
| **EMBASE** |  |  | **Cardiovascular diseases** |
|  |  | 1 | exp cardiovascular disease/ |
|  |  | 2 | exp heart disease/ |
|  |  | 3 | exp Coronary Disease/ |
|  |  | 4 | exp heart infarction/ |
|  |  | 5 | exp Myocardial Ischemia/ |
|  |  | 6 | exp angina pectoris/ |
|  |  | 7 | (isch?emi* adj3 heart).tw. |
|  |  | 8 | (myocard* adj3 (infarct* or re?vascular* or ischemi* or ischaem*)).tw. |
|  |  | 9 | (coronary adj3 disease*).tw. |
|  |  | 10 | ((coronary or cardiovascular or ischemic) adj event*).tw. |
|  |  | 11 | (heart adj (disease* or attack* or infarct*)).tw. |
|  |  | 12 | (cardiac adj3 disease).tw. |
|  |  | 13 | (morbid adj5 (heart* or cardiovascula* or coronary* or isch?em* or myocard*)).tw. |
|  |  | 14 | angina.tw. |
|  |  | 15 | MI.tw. |
|  |  | 16 | CHD or CAD.tw. |
|  |  | 17 | exp Stroke/ |
|  |  | 18 | (stroke or stokes or cerebrovasc* or cerebral vascular or apoplexy or (brain adj2 accident*)).tw. |
|  |  | 19 | (brain* or cerebral or lacunar) adj2 infarct*).tw. |
|  |  | 20 | or/1-19 |
|  |  |  | **Cardiovascular drugs** |
|  |  | 21 | exp Hydroxymethylglutaryl Coenzyme a Reductase Inhibitor/ |
|  |  | 22 | (HMG CoA reductase inhibitor*) or (HMG Co A reductase inhibitor*) |
|  |  | 23 | statin*.sh. |
|  |  | 24 | exp bile acid sequestrant/ |
|  |  | 25 | exp colesevelam/ |
|  |  | 26 | colestyramine/ |
|  |  | 27 | colestipol/ |
|  |  | 28 | ezetimibe/ |
|  |  | 29 | fibric acid derivative/ |
|  |  | 30 | nicotinic acid/ |
|  |  | 31 | (atorvastatin or cerivastatin or dalvastatin or fluindostatin or fluvastatin or lovastatin or pitavastatin or pravastatin or rosuvastatin or simvastatin or meglutol or mevinolin* or monacolin* or pravachol or lipex or lipitor or zocor or mevacor or lescol or baycol or bezafibrate or bezalip or ciprofibrate or modalim or clofibrate or fenofibrate or lipantil or supralip or gemfibrozil or lopid or procetofen or tocofibrte or transferal or theofibrate or etofylline clofibrate or duolip or acipimox or olbetam or nicotinic acid or niaspan).tw. |
|  |  | 32 | or/21-31 **[lipid modifiers]** |
|  |  | 33 | exp Antithrombocytic Agent/ |
|  |  | 34 | exp Phosphodiesterase Inhibitor/ |
|  |  | 35 | Defibrotide/ |
|  |  | 36 | platelet aggregation inhibit*.sh. |
|  |  | 37 | (antiplatelet agents* or anti-platelet agent*).sh. |
|  |  | 38 | (antiplatelet therap* or anti-platelet therap*).sh. |
|  |  | 39 | thrombocyte aggregation inhibit*.sh. |
|  |  | 40 | (antithrombocytic agent* or anti-thrombocytic agent*).sh. |
|  |  | 41 | (antithrombocytic therap* or anti-thrombocytic therap*).sh. |
|  |  | 42 | adenosine diphosphate receptor inhibit*.sh. |
|  |  | 43 | phosphodiesterase inhibit*.sh. |
|  |  | 44 | (adenosine reuptake inhibit* or adenosine re-uptake inhibit*).sh. |
|  |  | 45 | (aspirin or acetylsalicylic acid or dipyridamole or eptifibatide  or ticlopidine or  clopidogrel or cilostazol or (P2Y12 adj2 antagonis*) or prasugrel or cangrelor or ticagrelor or elinogrel tirofiban or picotamide or ticlid or beraprost or aggrenox or ditazole).sh. |
|  |  | 46 | or/33-45 **[Antiplatelet agents]** |
|  |  | 47 | exp thiazide diuretic agent/ |
|  |  | 48 | exp loop diuretic agent/ |
|  |  | 49 | exp Aldosterone Antagonist/ |
|  |  | 50 | (amiloride or benzothiadiazine or bendroflumethiazide or bumetanide or chlorothiazide or cyclopenthiazide or furosemide or hydrochlorothiazide or hydroflumethiazide or methyclothiazide or metolazone or polythiazide or trichlormethiazide or veratide or thiazide? or chlorthalidone or chlortalidone or phthalamudine or chlorphthalidolone or oxodoline or thalitone or hygroton or indapamide or metindamide or spironolactone* or eplerenone*).sh. |
|  |  | 51 | or/47-50 **[Diur]** |
|  |  | 52 | exp dipeptidyl carboxypeptidase inhibitor/ |
|  |  | 53 | angiotensin converting enzyme inhibit*.sh. |
|  |  | 54 | (alacepril or altiopril or ancovenin or benazepril or captopril or ceranapril or ceronapril or cilazapril or deacetylalacepril or delapril or derapril or enalapril or epicaptopril or fasidotril or fosinopril or foroxymithine or gemopatrilat or idapril or imidapril or indolapril or libenzapril or lisinopril or moexipril or moveltipril or omapatrilat or pentopril* or perindopril* or pivopril or quinapril* or ramipril* or rentiapril or saralasin or snitrosocaptopril or spirapril* or temocapril* or teprotide or trandolapril* or utibapril* or zabicipril* or zofenopril* or Aceon or Accupril or Altace or Capoten or Lotensin or Mavik or Monopril or Prinivil or Univas or Vasotec or Zestril).sh. |
|  |  | 55 | or/52-54 **[ACEI]** |
|  |  | 56 | exp renin inhibitor/ |
|  |  | 57 | (aliskiren or ciprokiren or ditekiren or enalkiren or remikiren or rasilez or tekturna or terlakiren or zankiren).sh. |
|  |  | 58 | renin inhibit*.sh. |
|  |  | 59 | or/56-58 **[RI]** |
|  |  | 60 | exp angiotensin receptor antagonist/ |
|  |  | 61 | (angiotensin adj3 (receptor antagon* or receptor block*)).sh. |
|  |  | 62 | (abitesartan or azilsartan or candesartan or elisartan or embusartan or eprosartan or forasartan or irbesartan or losartan ormilfasartan or olmesartan or saprisartan or tasosartan or telmisartan or valsartan or zolasartan).sh. |
|  |  | 63 | or/60-62 **[ARB]** |
|  |  | 64 | calcium channel blocking agent/ |
|  |  | 65 | (amlodipine or aranidipine or barnidipine or bencyclane or benidipine or bepridil or cilnidipine or cinnarizine or clentiazem or darodipine or diltiazem or efonidipine or elgodipine or etafenone or fantofarone or felodipine or fendiline or flunarizine or gallopamil or isradipine or lacidipine or lercanidipine or lidoflazine or lomerizine or manidipine or mibefradil or nicardipine or nifedipine or niguldipine or nilvadipine or nimodipine or nisoldipine or nitrendipine or perhexiline or prenylamine or semotiadil or terodiline or tiapamil or verapamil or Cardizem CD or Dilacor XR or Tiazac or Cardizem Calan or Isoptin or Calan SR or Isoptin SR Coer or Covera HS or Verelan PM).sh. |
|  |  | 66 | (calcium adj2 (antagonist? or block* or inhibit*)).sh. |
|  |  | 67 | or/64-66 **[CCB]** |
|  |  | 68 | exp beta adrenergic receptor blocking agent/ |
|  |  | 69 | (acebutolol or adimolol or afurolol or alprenolol or amosulalol or arotinolol or atenolol or befunolol or betaxolol or bevantolol or bisoprolol or bopindolol or bornaprolol or brefonalol or bucindolol or bucumolol or bufetolol or bufuralol or bunitrolol or bunolol or bupranolol or butofilolol or butoxamine or carazolol or carteolol or carvedilol or celiprolol or cetamolol or chlortalidone cloranolol or cyanoiodopindolol or cyanopindolol or deacetylmetipranolol or diacetolol or dihydroalprenolol or dilevalol or epanolol or esmolol or exaprolol or falintolol or flestolol or flusoxolol or hydroxybenzylpinodolol or hydroxycarteolol or hydroxymetoprolol or indenolol or iodocyanopindolol or iodopindolol or iprocrolol or isoxaprolol or labetalol or landiolol or levobunolol or levomoprolol or medroxalol ormepindolol ormethylthiopropranolol ormetipranolol ormetoprolol ormoprolol or nadolol or oxprenolol or penbutolol or pindolol or nadolol or nebivolol or nifenalol or nipradilol or oxprenolol or pafenolol or pamatolol or penbutolol or pindolol or practolol or primidolol or prizidilol or procinolol or pronetalol or propranolol or proxodolol or ridazolol or salcardolol or soquinolol or sotalol or spirendolol or talinolol or tertatolol or tienoxolol or tilisolol or timolol or tolamolol or toliprolol or tribendilol or xibenolol).sh. |
|  |  | 70 | or/68-79 **[BB]** |
|  |  | 71 | exp alpha adrenergic receptor blocking agent/ |
|  |  | 72 | (alfuzosin or bunazosin or doxazosin or metazosin or neldazosin or prazosin or silodosin or tamsulosin or terazosin or tiodazosin or trimazosin).sh. |
|  |  | 73 | ((andrenergic or alpha or receptor?) adj2 block*).sh. |
|  |  | 74 | or/72-74 **[AB]** |
|  |  | 75 | (51 or 55 or 59 or 63 or 67 or 70 or 74) |
|  |  | 76 | (32 and 46) or (32 and 75) or (46 and 75) |
|  |  | 77 | Evidence based.tw. |
|  |  | 78 | 76 or 77 |
|  |  |  | **Combination** |
|  |  | 79 | Drug Combinations/ |
|  |  | 80 | (polypill* or (drug* adj2 combin*) or ((multi* or several) adj2 (ingredient* or component* or therap* or treatment* or intervention*)) or policap or quintapill or (single adj2 pill* adj2 comb*) or single-pill or Red Heart pill*).tw. |
|  |  | 81 | (((mono* or single* or dual* or double* or triple*) adj3 (therap* or treatment* or intervention*)) or (intensive adj2 (lowing or reduction or management or therap* or treatment* or intervention*))).tw. |
|  |  | 82 | or/79-81 |
|  |  |  |  |
|  |  | 83 | 20 and 78 and 82 |
