# Supplementary material for: Effect of evidence-based therapy for secondary prevention of cardiovascular disease: Systematic review and meta-analysis
Source: PLoS One. 2019 Jan 18;14(1):e0210988. doi: 10.1371/journal.pone.0210988 (PMC6338367; doi:10.1371/journal.pone.0210988)
Supplement: S1 Table — (DOCX) [file pone.0210988.s003.docx]

| **Study** | **Participants** | **Outcomes** | **Intervention** | **Comparison** | **Adjusted results** | **Confounding variables adjusted for** | **Analysis method** |
| --- | --- | --- | --- | --- | --- | --- | --- |
| Al-Zakwani 2012 | ACS | 1-month mortality | EBCP: antiplatelet agent + ACEI/ARB + BB + ST | 0 EBC | OR 0.97 (0.71, 1.32) | Age, gender, family history of  CAD, diabetes mellitus, chronic renal failure, khat chewing, smoking status, heart rate, systolic blood pressure, Killip class score, ST-elevation myocardial infarction, GRACE risk score, in-hospital recurrent ischemia, inhospital re-infarction, in-hospital congestive heart failure, in-hospital cardiogenic shock, in-hospital stroke, in-hospital percutaneous coronary intervention and in-hospital coronary artery bypass graft. | Multivariable logistic regression |
|  |  | 1-year mortality |  |  | OR 1.20 (0.86, 1.67) |  |  |
| Amann 2014 | AMI | All-cause mortality | EBCP: antiplatelet agent + ACEI/ARB + BB + ST | Sub-EBCP | HR 0.63 (0.53, 0.74) | Age, gender, employment, smoking, type of AMI, reperfusion therapy, any in-hospital complication, history of stroke, diabetes, hyperlipidemia, and hypertension. | Multivariable Cox regression |
| Bauer 2010 | ACS | 1-year mortality | ≤3 EBCs of ASA, clopidogrel, ACEI/ARB, BB and ST | 4-5 EBCs | OR 1.6 (1.4, 1.9) | Age, gender, prior MI, prior stroke, smoking, hypercholesterolemia, hypertension, comorbidity, PCI and hospital bleeding. | Multivariable logistic regression models |
| Bezin 2017 | ACS | All-cause death or MACE | 1 EBC of Antiplatelet agents, ACEI/ARB, BB and ST | 4 EBCs | HR 1.76 (1.25, 2.48) | Age, gender, prior MI, prior drug use, prior medical consultations, patient status, characteristics of the initial ACS, comorbidities and use of CV drugs. | Cox regression |
|  |  |  | 2 EBCs |  | HR 1.54 (1.22, 1.94) |  |  |
|  |  |  | 3 EBCs |  | HR 1.25 (1.07, 1.47) |  |  |
| Bramlage 2010 | AMI | 1-year mortality | EBCP: ACEI/ARB + BB + ST + ASA + clopidogrel unless contraindicted | 0-1 EBC | OR 0.260 (0.179, 0.379) | Age, cardiac arrest on presentation, heart rate, systolic BP, Killip class, ST-segment deviation, abnormal cardiac biomarker, serum creatinine, previous MI and HF and in-hospital revascularization | Multivariable analysis (without clear description) |
|  |  |  | 2-4 EBCs |  | OR 0.486 (0.346, 0.684) |  |  |
|  |  |  | ST+ACEI/ARB+ASA+clopidogrel |  | OR 0.627 (0.339, 1.156) |  |  |
|  |  |  | ST+BB+ASA+clopidogrel |  | OR 0.248 (0.149, 0.411) |  |  |
|  |  |  | ST+ACEI/ARB+BB+ASA |  | OR 0.158 (0.034, 0.746) |  |  |
|  |  |  | ST+ACEI/ARB+BB+clopidogrel |  | OR 0.398 (0.254, 0.612) |  |  |
|  |  |  | ST+BB+ACEI/ARB |  | OR 0.482 (0.199, 1.170) |  |  |
|  |  |  | BB+ACEI/ARB+ASA+clopidogrel |  | OR 0.364 (0.228, 0.583) |  |  |
| Chen 2017 | CHD | All-cause mortality | EBCP: antiplatelet agents + ACEI/ARB + BB +ST | ≤ 2 EBCs | HR 0.60 (0.42, 0.87) | Age, sex, pre-hypertension, pre-diabetes, current smoker, BMI, ACS, nation, revascularization, marital status, previous MI, previous PCI, OMT before admission, serum creatinine, glucose, triglyceride, low-density lipoprotein, white blood cell, platelet and systolic BP. | Multivariable Cox regression |
|  |  |  | 3 EBCs |  | HR 0.76 (0.54, 1.08) |  |  |
|  | ACS | All-cause mortality | EBCP |  | HR 0.62 (0.40, 0.96) |  |  |
|  |  |  | 3 EBCs |  | HR 0.85 (0.56, 1.3) |  |  |
|  | Stable angina | All-cause mortality | EBCP |  | HR 0.45 (0.22, 0.91) |  |  |
|  |  |  | 3 EBCs |  | HR 0.42 (0.21, 0.84) |  |  |
| Danchin 2005 | AMI | 1-year mortality | EBCP: Antiplatelet agents + BB + ST | ≤ 2 EBCs | HR 0.52 (0.33, 0.81) | Age, sex, history of hypertension, current smoking, history of CVD, admission systolic BP and heart rate, use of reperfusion therapy, LVEF, Killip class, atrial fibrillation, atrioventricular block, PCI, use of diuretics, digitalis, nitrate, triple combination therapy and propensity score. | Multivariable Cox regression; propensity score |
| Gouya 2007 | AMI | All-cause mortality | ≤ 2 EBCs of antiplatelet agents, ACEI/ARB, BB and lipid modifiers | 3-4 EBCs | HR 1.64 (0.86, 3.1) | Age and sex | Cox regression |
| Kopel 2014 | AMI | 1-year mortality | 3-4 EBCs of ASA, ACEI/ARB, BB and ST | 0-2 EBCs | HR 0.66 (0.50, 0.87) | Baseline, admission presentation, in-hospital course variables, pre-admission drug and propensity score | Cox regression |
| Lafeber 2013 | CAD | All-cause mortality | EBCP: ASA + ST + BP-lowering agents | Sub-EBCP | HR 0.69 (0.49, 0.96) | Age, gender, BMI, smoking, pack-years of smoking, presence of concomitant vascular disease (CVD, PAOD, AAA), total cholesterol, HDL cholesterol, and systolic BP. | Cox regression; propensity score |
|  |  | MI |  |  | HR 0.68 (0.49, 0.96) |  |  |
|  |  | Ischemic cerebrovascular accident |  |  | HR 0.37 (0.16, 0.84) |  |  |
|  |  | Composite vascular outcome |  |  | HR 0.66 (0.49, 0.88) |  |  |
|  |  | Vascular mortality |  |  | HR 0.53 (0.33, 0.85) |  |  |
|  |  | All-cause mortality | 2 EBCs | 3 EBCs | HR 2.07 (1.69, 2.53) |  |  |
|  |  | MI |  |  | HR 1.44 (1.13, 1.83 |  |  |
|  |  | Ischemic cerebrovascular accident |  |  | HR 1.59 (109, 2.32) |  |  |
|  |  | Composite vascular outcome |  |  | HR 1.50 (1.24, 1.81) |  |  |
|  |  | Vascular mortality |  |  | HR 1.97 (1.52, 2.55) |  |  |
|  |  | All-cause mortality | 1 EBC | 3 EBCs | HR 2.23 (1.79, 2.78) |  |  |
|  |  | MI |  |  | HR 1.53(1.16, 2.01) |  |  |
|  |  | Ischemic cerebrovascular accident |  |  | HR 1.46 (0.96, 2.23) |  |  |
|  |  | Composite vascular outcome |  |  | HR 1.62 (1.32, 2.00) |  |  |
|  |  | Vascular mortality |  |  | HR 2.30 (1.74, 3.04) |  |  |
| Lee 2010 | AMI | 6-month mortality | EBCP: antiplatelet agents + ACEI/ARB + BB +ST | 2-3 EBCs | HR 0.394 (0.161, 0.963) | Age, anterior MI, Killip class, LVEF, serum creatinine levels and multivessel disease. | Cox regression |
|  |  |  |  | 0-1 EBC | HR 0.488 (0.205, 1.165) |  |  |
| Mukherjee 2004 | ACS | 6-month mortality | Level IV of EBCP | 0 EBC | OR 0.10 (0.03, 0.42) | Age, gender, positive biomarker, new ST elevation, left ventricular ejection fraction, history of diabetes, renal failure, heart failure, and revascularization. | Multivariable logistic regression |
|  |  |  | Level III |  | OR 0.17 (0.04, 0.75) |  |  |
|  |  |  | Level II |  | OR 0.18 (0.04, 0.77) |  |  |
|  |  |  | Level I |  | OR 0.36 (0.08, 1.75) |  |  |
| Park 2015 | ACS | All-cause death | EBCP: Antihypertensive agents + lipid modifiers + antithrombotic agents | 0 EBC | HR 0.35 (0.13, 0.96) | age, sex, ethnicity, hypertension, diabetes, smoking, history of CHD, history of carotid endarterectomy, systolic BP, BMI, low-density lipoprotein cholesterol, triglyceride, and high-density lipoprotein cholesterol level | Cox regression |
|  |  |  | 2 EBCs |  | HR 0.71 (0.26, 1.93) |  |  |
|  |  |  | 1 EBC |  | HR 0.89 (0.30, 2.64) |  |  |
|  |  | Stroke | 3 EBCs |  | HR 0.39 (0.18, 0.84) |  |  |
|  |  |  | 2 EBCs |  | HR 0.50 (0.23, 1.09) |  |  |
|  |  |  | 1 EBC |  | HR 0.51 (0.21, 1.25) |  |  |
|  |  | Stroke/CHD/vascular death | 3 EBCs |  | HR 0.39 (0.22, 0.69) |  |  |
|  |  |  | 2 EBCs |  | HR 0.45 (0.25, 0.80) |  |  |
|  |  |  | 1 EBC |  | HR 0.60 (0.32, 1.14) |  |  |
| Tay 2008 Younger cohort | AMI | 1-year mortality | EBCP: Antiplatelet agents + ACEI/ARB +BB + lipid-modifiers | 0 EBC | OR 0.03 (0.02, 0.16) | Age, sex, race, smoking, dyslipidemia, hypertension, diabetes mellitus, history of AMI, prior PCI/coronary artery bypass grafting, revascularisation, and Killip class. | Multivariable logistic regression model |
|  |  |  | 3 EBCs |  | OR 0.05 (0.03, 0.09) |  |  |
|  |  |  | 2 EBCs |  | OR 0.10 (0.06, 0.17) |  |  |
|  |  |  | 1 EBC |  | OR 0.28 (0.16, 0.50) |  |  |
| Elderly cohort | AMI | 1-year mortality | 4 EBCs | 0 EBC | OR 0.10 (0.05, 0.21) |  |  |
|  |  |  | 3 EBCs |  | OR 0.16 (0.08, 0.31) |  |  |
|  |  |  | 2 EBCs |  | OR 0.18 (0.09, 0.35) |  |  |
|  |  |  | 1 EBC |  | OR 0.44 (0.22, 0.87) |  |  |
| Timoteo  2006 | ACS | 30-days mortality | 3-4 EBCs | 1-2 EBCs | OR 0.23 (0.11, 0.48) | Unclear | Kaplan-Meier curves, log-rank test |
| Yan 2007 | ACS | 1-year mortality | EBCP: antiplatelet/anticoagulant + ACEI + BB + lipid-modifiers | 0-1 EBC | OR 0.54 (0.36, 0.81) | Global Registry of Acute Cardiac Events (GRACE) risk score: adjusted age, cardiac arrest on presentation, heart rate, systolic BP, Killip class, ST-segment deviation, abnormal cardiac biomarker, and serum creatinine. | Multivariable logistic regression |
|  |  |  | 2-3 EBCs | 0-1 EBC | OR 0.65 (0.47, 0.90) |  |  |
| Zeymer 2011 | AMI patients treated with BB | 1-year mortality | 2 EBCs | ASA+ACEI+ST | OR 1.54 (1.26, 1.87) | Propensity score | Multivariable logistic regression models; propensity score |
|  |  |  | 0-1 EBC |  | OR 1.67 (1.24, 2.27) |  |  |
|  |  | MACCE | 2 EBCs |  | OR 1.27 (1.08, 1.49) |  |  |
|  |  |  | 0-1 EBC |  | OR 1.49 (1.14, 1.95) |  |  |
| Hippisley 2005 | IHD | All-cause mortality | ST | 0 EBC | OR 0.53 (0.33, 0.86) | Comorbidity (diabetes, hypertension, congestive cardiac failure, and MI), use of CCB, smoking status, BMI, and Townsend score | Conditional logistic regression |
|  |  |  | ACEI |  | OR 0.80 (0.65, 0.99) |  |  |
|  |  |  | ASA |  | OR 0.59 (0.50, 0.68) |  |  |
|  |  |  | BB |  | OR 0.81 (0.63, 1.04) |  |  |
|  |  |  | ST+ASA |  | OR 0.39 (0.29, 0.52) |  |  |
|  |  |  | ST+BB |  | OR 0.46 (0.26, 0.82) |  |  |
|  |  |  | ACEI+ASA |  | OR 0.54 (0.45, 0.66) |  |  |
|  |  |  | ACEI+BB |  | OR 0.64 (0.43, 0.94) |  |  |
|  |  |  | ASA+BB |  | OR 0.38 (0.31, 0.47) |  |  |
|  |  |  | ST+ACEI+BB |  | OR 0.29 (0.21, 0.41) |  |  |
|  |  |  | ST+ACEI+BB |  | OR 0.67 (0.30, 1.51) |  |  |
|  |  |  | ST+ASA+BB |  | OR 0.17 (0.12, 0.23) |  |  |
|  |  |  | ACEI+ASA+BB |  | OR 0.34 (0.26, 0.46) |  |  |
|  |  |  | ST+ACEI+ASA+BB |  | OR 0.25 (0.18, 0.35) |  |  |
| Kirchmayer 2013 | AMI | All-cause mortality | EBCP: Eantiplatelet agents + ACEI/ARB + BB + ST | 0 EBC | OR 0.35 (0.21, 0.59) | PCI and bypass at index admission, HF, malignant neoplasm, disorders of lipoid metabolism/obesity, diabetes, chronic nephropathies, cerebrovascular disease, diseases of arteries, arterioles and capillaries, hemorrhagic stroke, hematologic diseases, cardiac dysrhythmias, duration of the index admission. | Conditional logistic regression |
|  |  |  | 3 EBCs |  | OR 0.59 (0.46, 0.76) |  |  |
|  |  |  | 2 EBCs |  | OR 0.59 (0.47, 0.76) |  |  |
|  |  |  | 1 EBC |  | OR 0.68 (0.53, 0.87) |  |  |
|  |  | Reinfarction | EBCP: antiplatelet agents + ACEI/ARB + BB + ST | 0 EBC | OR 0.23 (0.15, 0.37) | PCI and bypass at index admission, HF, diabetes, chronic nephropathies, diseases of arteries, arterioles and capillaries, ACEIs/sartans before admission, duration of the index admission. |  |
|  |  |  | 3 EBCs |  | OR 0.37 (0.28, 0.47) |  |  |
|  |  |  | 2 EBCs |  | OR 0.49 (0.38, 0.62) |  |  |
|  |  |  | 1 EBC |  | OR 0.73 (0.57, 0.97) |  |  |
| Van 2007 | MI | Recurrent MI | 3 EBCs of antiplatelet agents, ACEI/BB and ST | 0 EBC | OR 0.59 (0.37, 0.94) | diabetes mellitus, angina, use of anticoagulants, antiarrhythmic drugs, digoxin and CCBs, admission for chronic HF and PTCA or coronary artery bypass grafting procedure between first MI and index date | Conditional logistic regression |
|  |  |  | 2 EBCs |  | OR 0.74 (0.53, 1.03) |  |  |
|  |  |  | 1 EBC |  | OR 0.94 (0.70, 1.28) |  |  |

Abbreviations: AAA = abdominal aortic aneurysm; ACEI = angiotensin-converting enzyme inhibitor; ACS = acute coronary syndromes; AMI = acute myocardial infarction; ARB = angiotensin receptor blocker; ASA = acetyl salicylic acid; BB = beta-blockers; BMI = body mass index; BP = blood pressure; CAD = coronary artery disease; CCB = calcium channel blockers; CV = cardiovascular; EBCP = evidence-based combination pharmacotherapy; EBC = evidence-based component; GRACE =Global Registry of Acute Coronary Events; HDL = high-density lipoprotein; HF = heart failure; HR = hazard ratio; LVEF = left ventricular ejection fraction; MACE = major adverse cardiac event; MACCE = major adverse cardiovascular and cerebrovascular event; MI = myocardial infarction; OMT = optimal medical therapy; OR = odds ratio; PAOD = peripheral arterial occlusive disease; PCI = percutaneous coronary intervention; PTCA = percutaneous transluminal coronary angioplasty; RR = risk ratio; ST = statins
