# Supplementary material for: Effect of evidence-based therapy for secondary prevention of cardiovascular disease: Systematic review and meta-analysis
Source: PLoS One. 2019 Jan 18;14(1):e0210988. doi: 10.1371/journal.pone.0210988 (PMC6338367; doi:10.1371/journal.pone.0210988)
Supplement: S2 Table — (DOCX) [file pone.0210988.s004.docx]

| **Study^*^** | **Selection** | | | | **Comparability of cohort** | **Outcome** | | | **Overall quality** |
| --- | --- | --- | --- | --- | --- | --- | --- | --- | --- |
|  | **Exposed cohort representative** | **Non-exposed cohort selection** | **Exposure ascertainment** | **Outcome not present at start** |  | **Assessment** | **Follow-up length** | **Follow-up adequacy** |  |
| Al-Zakwani 2012 | ***** | ***** | ***** | ***** | ****** | ***** | ***** | ***** | **9** |
| Amann 2014 | ***** | ***** | ***** | ***** | ****** | ***** | ***** | ***** | **9** |
| Bauer 2010 | ***** | ***** | ***** | ***** | ****** | ***** | ***** | ***** | **9** |
| Bezin 2017 | ***** | ***** | ***** | ***** | ****** | ***** | ***** | ***** | **9** |
| Bramlage 2010 | ***** | ***** | ***** | ***** | ****** | ***** | ***** | ***** | **9** |
| Chen 2017 | **-** | ***** | ***** | ***** | ****** | ***** | ***** | ***** | **8** |
| Danchin 2005 | ***** | ***** | ***** | ***** | ****** | ***** | ***** | ***** | **9** |
| Gouya 2007 | ***** | ***** | ***** | ***** | ***** | ***** | ***** | ***** | **8** |
| Gunnell 2013 | ***** | ***** | ***** | ***** | ****** | **-** | ***** | ***** | **8** |
| Kopel 2014 | ***** | ***** | ***** | ***** | ****** | ***** | ***** | ***** | **9** |
| Lafeber 2013 | ***** | ***** | ***** | **-** | ****** | ***** | ***** | ***** | **8** |
| Lee 2010 | ***** | ***** | ***** | ***** | ****** | ***** | **-** | ***** | **8** |
| Mukherjee2004 | **-** | ***** | ***** | ***** | ****** | ***** | ***** | ***** | **8** |
| Park 2015 | ***** | ***** | ***** | **-** | ****** | ***** | ***** | **-** | **7** |
| Tay 2008 | **-** | ***** | ***** | ***** | ****** | ***** | ***** | ***** | **8** |
| Timoteo2006 | **-** | ***** | ***** | ***** | **-** | ***** | ***** | **-** | **5** |
| Yan 2007 | ***** | ***** | ***** | ***** | ****** | ***** | ***** | ***** | **9** |
| Zeymer 2011 | ***** | ***** | ***** | ***** | ****** | ***** | ***** | ***** | **9** |

^*^Newcastle-Ottawa Quality Assessment Scale: 1 star (*) for meeting each criterion, except comparability (design or analysis) can have 2 stars. For comparability in this review: 1 star if controlled for age; 2 stars if also controlled for other important variables, e.g., exercise, body mass index, use of hormone replacement therapy or other relevant drugs
