# Supplementary material for: Effect of evidence-based therapy for secondary prevention of cardiovascular disease: Systematic review and meta-analysis
Source: PLoS One. 2019 Jan 18;14(1):e0210988. doi: 10.1371/journal.pone.0210988 (PMC6338367; doi:10.1371/journal.pone.0210988)
Supplement: S3 Table — (DOCX) [file pone.0210988.s005.docx]

| **Study^*^** | **Selection** | | | | **Comparability of cases and controls** | **Exposure** | | | **Overall quality** |
| --- | --- | --- | --- | --- | --- | --- | --- | --- | --- |
|  | **Case definition** | **Cases representative** | **Control selection** | **Control definition** |  | **Ascertainment method** | **Same ascertainment both groups** | **Non-response rate** |  |
| Hippisley 2005 | **-** | ***** | ***** | ***** | ****** | **-** | ***** | ***** | 7 |
| Kirchmayer 2013 | **-** | ***** | ***** | ***** | ****** | **-** | ***** | ***** | 7 |
| Van 2007 | **-** | ***** | ***** | ***** | ****** | **-** | ***** | ***** | 7 |

^*^Newcastle-Ottawa Quality Assessment Scale: 1 star (*) for meeting each criterion, except comparability (design or analysis) can have 2 stars. For comparability in this review: 1 star if controlled for age; 2 stars if also controlled for other important variables, e.g., exercise, body mass index, use of hormone replacement therapy or other relevant drugs
