# Supplementary material for: Effect of evidence-based therapy for secondary prevention of cardiovascular disease: Systematic review and meta-analysis
Source: PLoS One. 2019 Jan 18;14(1):e0210988. doi: 10.1371/journal.pone.0210988 (PMC6338367; doi:10.1371/journal.pone.0210988)
Supplement: S4 Table — (DOCX) [file pone.0210988.s006.docx]

| **Studies excluded** | **Random effects model** | | **Test of heterogeneity** | | |  |
| --- | --- | --- | --- | --- | --- | --- |
|  | **RR** | **95% CI** | | **I^2^, %** | **P-value** | |
| None | 0.60 | 0.55, 0.66 | | 87.9 | <0.01 | |
| Tay 2008 | 0.68 | 0.64, 0.72 | | 72.1 | <0.01 | |
| Hippisley 2005 | 0.57 | 0.50, 0.65 | | 89.4 | <0.01 | |
| Kirchmayer 2013 | 0.59 | 0.54, 0.65 | | 87.7 | <0.01 | |
| Yan 2007 | 0.59 | 0.54, 0.65 | | 88.0 | <0.01 | |

Abbreviations: RR = Relative Risk; CI = Confidence Interval
