# Supplementary material for: Effect of evidence-based therapy for secondary prevention of cardiovascular disease: Systematic review and meta-analysis
Source: PLoS One. 2019 Jan 18;14(1):e0210988. doi: 10.1371/journal.pone.0210988 (PMC6338367; doi:10.1371/journal.pone.0210988)
Supplement: S5 Table — (DOCX) [file pone.0210988.s007.docx]

| **Covariate** | **Classification** | **No of studies or subsets** | **Risk ratio of subgroup analysis (95% CI)** | **Tests for** **heterogeneity** | | **P value of subgroup difference** |
| --- | --- | --- | --- | --- | --- | --- |
|  |  |  |  | **P value** | **I^2^(%)** |  |
| **Age** | <65 years | 9 | 0.44 (0.30, 0.63) | < 0.01 | 94.0 | 0.01 |
|  | 65-75 years | 15 | 0.71 (0.65, 0.77) | < 0.01 | 57.7 |  |
|  | >75 years | 14 | 0.62 (0.56, 0.69) | < 0.01 | 84.7 |  |
| **Region** | Multi-region | 2 | 0.74 (0.54, 1.01) | 0.328 | 0.0 | < 0.01 |
|  | Asia | 8 | 0.40 (0.31, 0.53) | < 0.01 | 86.3 |  |
|  | Europe | 23 | 0.67 (0.63, 0.72) | < 0.01 | 76.6 |  |
|  | Canada/USA | 5 | 0.65 (0.51, 0.83) | 0.017 | 66.7 |  |
| **Disease** | Stroke | 2 | 0.74 (0.54, 1.01) | 0.328 | 0.0 | 0.16 |
|  | ACS | 5 | 0.65 (0.51, 0.83) | 0.017 | 66.7 |  |
|  | CHD | 12 | 0.67 (0.61, 0.73) | < 0.01 | 82.3 |  |
|  | AMI | 19 | 0.54 (0.46, 0.64) | < 0.01 | 91.6 |  |
| **Follow-up** | <1 year | 2 | 0.57 (0.44, 0.73) | 0.264 | 23.7 | 0.02 |
|  | 1 year | 16 | 0.52 (0.43, 0.64) | < 0.01 | 92.0 |  |
|  | >1 year | 17 | 0.69 (0.63, 0.74) | < 0.01 | 80.1 |  |
| **Study type** | Retrospective cohort study | 2 | 0.74 (0.54, 1.01) | 0.328 | 0.0 | 0.02 |
|  | Prospective cohort study | 22 | 0.54 (0.46, 0.63) | < 0.01 | 89.8 |  |
|  | Case-control study | 14 | 0.68 (0.62, 0.75) | < 0.01 | 87.9 |  |
| Abbreviations: ACS = Acute Coronary Syndrome; AMI = Acute Myocardial Infarction; CHD = Coronary Heart Disease; CI = Confidence Interval; USA = the United States of America | | | | | |  |
