# Supplementary figures and images for: Effect of evidence-based therapy for secondary prevention of cardiovascular disease: Systematic review and meta-analysis
Source: PLoS One. 2019 Jan 18;14(1):e0210988. doi: 10.1371/journal.pone.0210988 (PMC6338367; doi:10.1371/journal.pone.0210988)

**
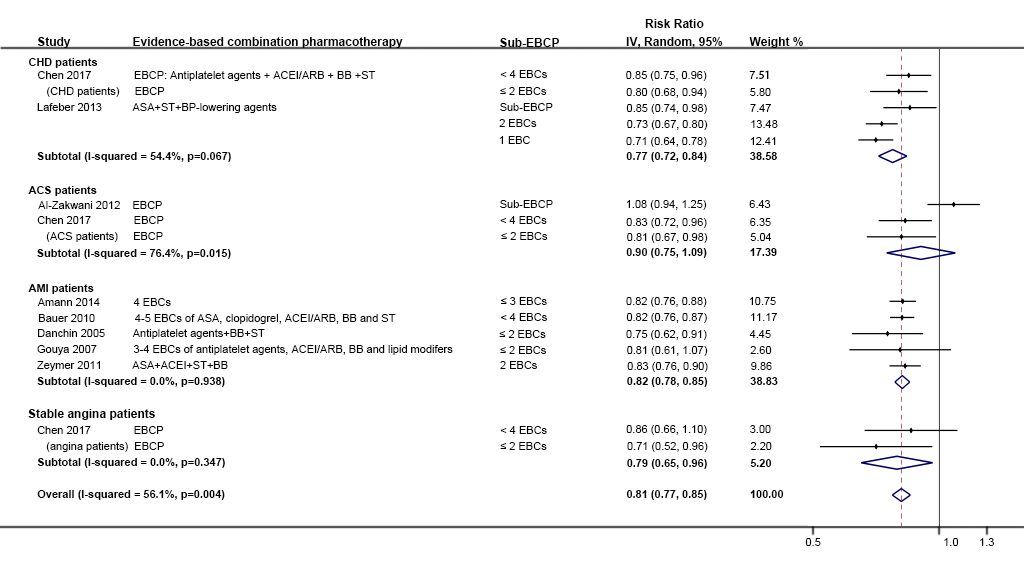
**

Supplement: S1 Fig — (DOCX) [file pone.0210988.s008.docx]

**
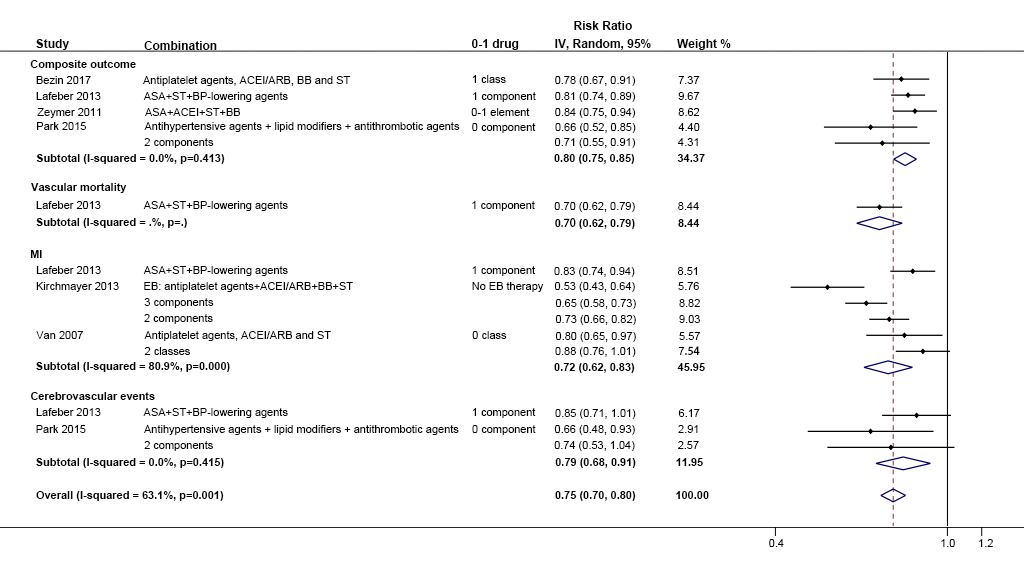
**

Supplement: S2 Fig — (DOCX) [file pone.0210988.s009.docx]

**
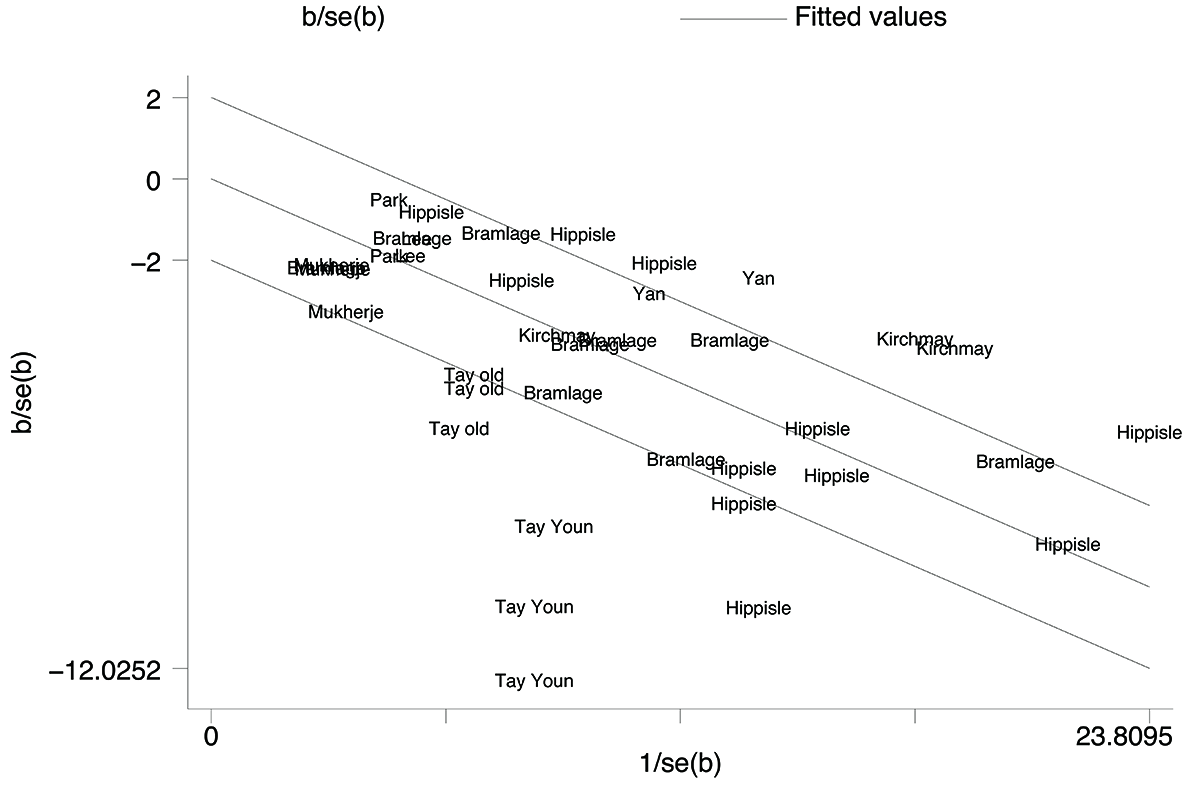
**

Supplement: S3 Fig — (DOCX) [file pone.0210988.s010.docx]

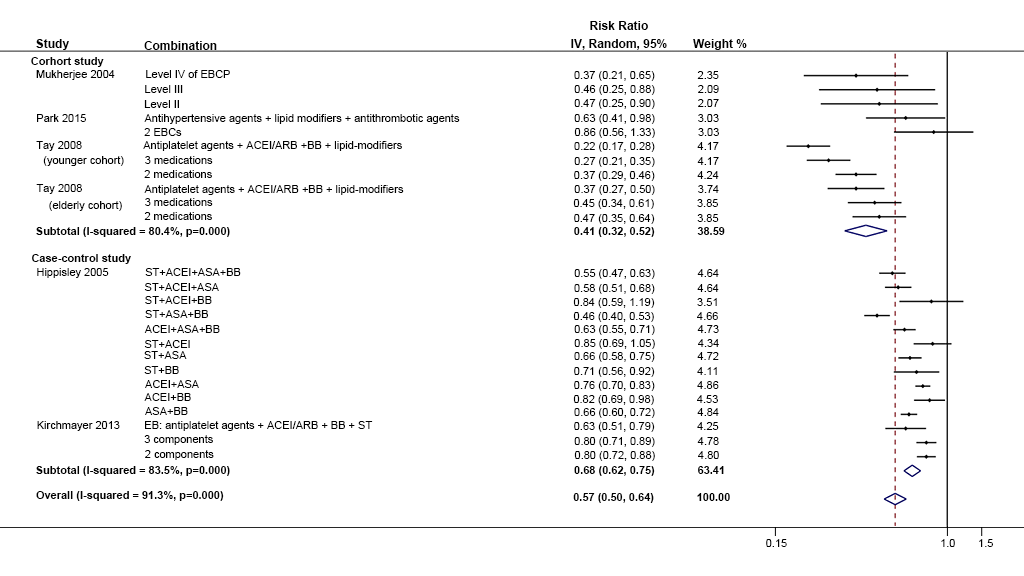

Supplement: S4 Fig — (DOCX) [file pone.0210988.s011.docx]
